# Targeted discovery of gut microbiome-remodeling compounds for the treatment of systemic inflammatory response syndrome

**DOI:** 10.1128/msystems.00788-24

**Published:** 2024-09-05

**Authors:** Luyao Liu, Lin Ma, Huan Liu, Fan Zhao, Pu Li, Junhua Zhang, Xin Lü, Xin Zhao, Yanglei Yi

**Affiliations:** 1 College of Food Science and Engineering, Northwest A&F University, Shaanxi, China; 2 State Key Laboratory of Component-based Chinese Medicine, Tianjin University of Traditional Chinese Medicine, Tianjin, China; 3 Department of Emergency, Ruijin Hospital, Shanghai Jiao Tong University School of Medicine, Shanghai, China; 4 College of Animal Science and Technology, Northwest A&F University, Shaanxi, China; 5 Department of Critical Care Medicine, The Second Affiliated Hospital of Air Force Medical University, China, Shaanxi; Politecnico di Torino, Turin, Italy

**Keywords:** gut microbiome remodeling, *in vitro *screening, systemic inflammation response syndrome, gut inflammation

## Abstract

Systemic inflammatory response syndrome (SIRS) is a severe inflammatory response that can lead to organ dysfunction and death. Modulating the gut microbiome is a promising therapeutic approach for managing SIRS. This study assesses the therapeutic potential of the Xuanfei Baidu (XFBD) formula in treating SIRS. The results showed that XFBD administration effectively reduced mortality rates and inflammation in SIRS mice. Using 16S rRNA sequencing and fecal microbiota transplantation (FMT), we substantiated that the therapeutic effects of XFBD are partly attributed to gut microbiota modulation. We conducted *in vitro* experiments to accurately assess the gut microbiome remodeling effects of 51 compounds isolated from XFBD. These compounds exhibited varying abilities to induce a microbial structure that closely resembles that of the healthy control group. By quantifying their impact on microbial structure and clustering their regulatory patterns, we devised multiple gut microbiome remodeling compound (GMRC) cocktails. GMRC cocktail C, comprising aucubin, gentiopicroside, syringic acid, gallic acid, p-hydroxybenzaldehyde, para-hydroxybenzoic acid, and isoimperatorin, demonstrated superior efficacy in treating SIRS compared to a single compound or to other cocktails. Finally, *in vitro* experiments showcased that GMRC cocktail C effectively rebalanced bacteria composition in SIRS patients. This study underscores XFBD’s therapeutic potential in SIRS and highlights the importance of innovative treatment approaches for this disease by targeting the gut microbiota.

**IMPORTANCE:**

Developing effective treatment strategies for systemic inflammatory response syndrome (SIRS) is crucial due to its severe and often life-threatening nature. While traditional treatments like dexamethasone have shown efficacy, they also come with significant side effects and limitations. This study makes significant strides by demonstrating that the Xuanfei Baidu (XFBD) formula can substantially reduce mortality rates and inflammation in SIRS mice through effective modulation of the gut microbiota. By quantitatively assessing the impact of 51 compounds derived from XFBD on the gut microbiome, we developed a potent gut microbiome remodeling compound cocktail. This cocktail outperformed individual compounds and other mixtures in efficacy against SIRS. These findings highlight the potential of XFBD as a therapeutic solution for SIRS and underscore the critical role of innovative strategies targeting the gut microbiota in addressing this severe inflammatory condition.

## INTRODUCTION

The gut microbiome is a diverse and complex ecosystem, inhabited by thousands of microbial species that coevolved within the host and actively impacted multiple host functions (1). The normal intestinal microbiota plays a major role in the maintenance of health and disease prevention (2). It confers protection from pathogenic organisms that cause infection, facilitates digestion of complex plant carbohydrates into short-chain fatty acids (SCFAs), produces small molecules that interact with the host environment, and maintains cell homeostasis. It also shapes the development of the immune system (3–6). Several studies suggest that functional/compositional alterations in gut microbiota may contribute to the inflammation by influencing differentiation of inflammatory cell types, cytokine production, and hematopoiesis (7). Systemic inflammatory response syndrome (SIRS) is caused by a defense response of the body to a noxious stressor to localize and then eliminate the endogenous or exogenous source of the insult (8). When SIRS occurs as a result of infection, it is termed sepsis. Even though the purpose is defensive, the dysregulated cytokine storm can cause a massive inflammatory cascade leading to reversible or irreversible end-organ dysfunction and even death. For instance, SIRS derived from lung injury caused by SARS-CoV-2 infection may result in severe outcomes in COVID-19 (9). SIRS is characterized by dysregulated cytokine release, immune responses and is associated with organ dysfunction (10). It has been shown that patients with sepsis have a profoundly distorted composition of the intestinal microbiota. The microbiota of intensive care unit (ICU) patients with sepsis has an increased abundance of microbes tightly associated with inflammation, such as *Parabacteroides*, *Fusobacterium*, and *Bilophila* species (11). Moreover, patients with sepsis have impaired gastrointestinal motility and diminished intestinal epithelial integrity, leading to a loss of “beneficial” anaerobic bacterial families, such as *Lachnospiraceae* and *Ruminococcaceae*, which further impairs intestinal epithelium function and allows for the expansion and potential translocation of aerobic opportunistic pathogens (12).

The role of the gut microbiome is increasingly understood during SIRS/sepsis. Gut microbiome modulation, aiming to restore gut microbial homeostasis, has become a therapeutic option in the treatment of SIRS and sepsis. Selective decontamination of the digestive tract has been implemented in many ICUs to prevent nosocomial infections and decrease overall mortality rates (13, 14). Fecal microbiota transplantation (FMT) involves the transfer of feces in its entirety from one individual to another to attempt to repopulate the gut with healthy microbiota (15). Several case reports have noted a decrease in SIRS response including fever in patients in the intensive care setting following FMT (16, 17). In addition, live biotherapeutics-single or multi-probiotic strains, have been investigated extensively and are recently offered as a means to prevent sepsis in patients undergoing elective gastrointestinal surgery (18, 19). Given the sensitivity (plasticity) of microbiota to the changes in the chemical milieu within the gut, the microbiome can be manipulated therapeutically by small molecules and drugs as well.

Small-molecule drugs offer new opportunities and have emerged as a new frontier for gut microbiome remodeling compounds (GMRCs) discovery and precision medicine. Several promising small molecules that can modulate the microbiome are being developed to treat various diseases including inflammatory bowel disease, Parkinson’s disease, hyperammonaemia, and so on (20). The current discovery paradigm of GMRCs continues to follow a conventional “one-drug-one-test” process, which constantly needs to progress, increasing efficiency and reducing time to market. Recent advances in high-throughput approaches for the cultivation of fastidious anaerobes allowed systematic studies of the effects of drugs on gut microbiome (21). The *in vitro* approach has proven to be advantageous in terms of providing a systematic, controlled, and question-specific analysis of the interaction between drugs and the gut microbiome, which can be studied at different levels of scale (21–23).

Xuanfei Baidu (XFBD) formula is a first-line traditional Chinese medicine (TCM) formula used for COVID-19 patients with symptoms of “Shi Du Yu Fei'” (the lung is stagnated by noxious dampness). In addition, XFBD protects against macrophage-induced inflammation and pulmonary fibrosis via inhibiting IL-6/STAT3 signaling pathway (24). Our previous data indicated that XFBD treatment attenuated intestinal disorders associated with inhibiting inflammation, remodeling of intestinal immunity, and improving intestinal flora (25). XFBD is composed of 13 medicinal plants with more than 100 active compounds. Determining the effects of each individual compound is crucial to understanding the mechanism of XFBD and also presents a significant obstacle in the field of traditional medical research. On the other hand, the complex nature of TCM with proven clinical efficacy are notable reservoir for GMRC discovery. For instance, Pien Tze Huang (PZH) modulates gut microbiota and metabolites, exhibiting anti-inflammatory and anticancer properties, ultimately suppressing colorectal carcinogenesis (26).

This study investigated the therapeutic effects of XFBD on SIRS using a lipopolysaccharide (LPS) induced mouse model. Our results suggest that XFBD regulates gut microbiota as one of its significant mechanisms in treating SIRS. Furthermore, an *in vitro* approach was implemented for the targeted screening of GMRCs that could remodel the gut microbiome of SIRS mice. Based on our quantitative *in vivo* study, we conducted a thorough screening process to identify potential GMRCs and subsequently developed various GMRC cocktails. We evaluated the effectiveness of these cocktails in treating SIRS and found that they were effective. Our findings highlight the utility of *in vitro* screening for rapidly identifying effective GMRCs and underscore the therapeutic potential of GMRC cocktails derived from TCM formulas for treating SIRS. Moreover, our research highlights the significance of rigorous *in vitro* screening processes in identifying effective treatments for complex health conditions.

## RESULTS

### XFBD alleviates LPS-induced SIRS in mice

**Fig 1:**
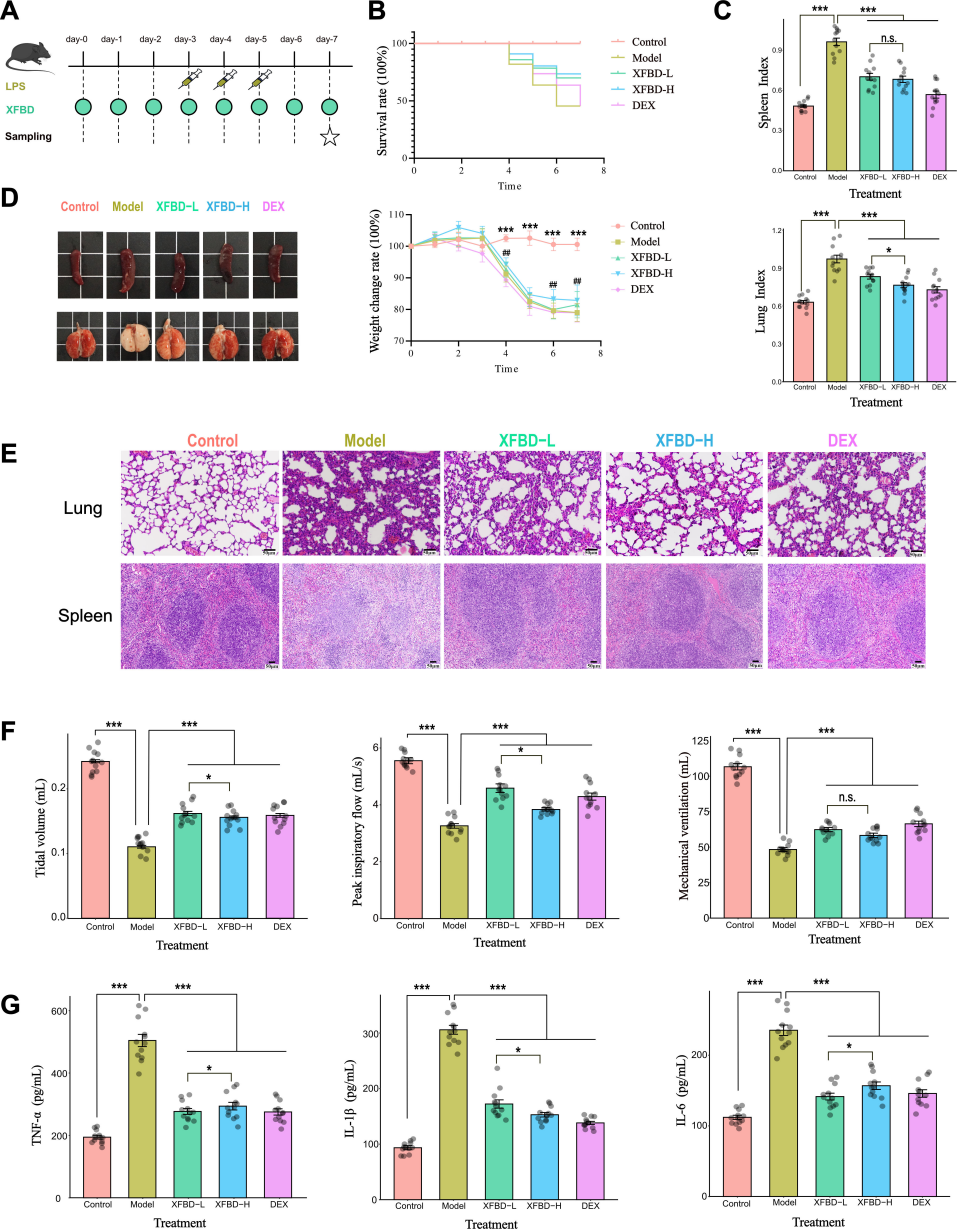
Alleviation effects of LPS-induced systemic inflammatory response syndrome (SIRS) in mice by XFBD treatment. (**A**)bExperimental flowchart illustrating the study design and procedures. (**B**) Survival curves and daily body weight changes from days 0 to 7 (*n* = 12 per group). Statistical significance were determined using one-way ANOVA and two-way ANOVA respectively, followed by Tukey test. *P ≤ 0.05, **P ≤ 0.01, ***P ≤ 0.001 was model relative to control group, #*P* ≤ 0.05, ##*P* ≤ 0.01, ###*P* ≤ 0.001 was XFBD-H relative to model group. (**C**) Spleen and lung weight index of each group (*n* = 12 per group). (**D**) Organ morphology of representative spleens and lungs of mice from different groups (*n* = 6 per group). (**E**) H&E staining of lung and spleen tissue sections (*n* = 6 per group). (**F**) Lung function results of tidal volume (TV), peak inspiratory flow (PIF), and minute volume (MV) (*n* = 12 per group). (**G**) The serum concentrations of TNF-α, IL-1β, and IL-6 in mice from different groups (*n* = 12 per group). Data were presented as mean ± SEM (*n* = 12 per group). Statistical significance was determined using one-way ANOVA, followed by Tukey test. n.s. not significant, *P < 0.05, **P < 0.01, ***P < 0.001.

The LPS-induced SIRS model was utilized to investigate the therapeutic effect of XFBD on SIRS. XFBD was administered to the experimental animals to observe its efficacy in reducing the inflammation associated with SIRS (Fig. 1A). Compared with the control group, the survival rate decreased in the LPS-induced model group. The results suggested that XFBD could effectively improve the survival rate (Fig. 1B). Activation of the immune system releases inflammatory mediators, leads to disruption of physiological functions and organ dysfunction, of which the lungs are the most sensitive (27). Compared with the control group, the spleen and lung indices of the model group were significantly increased, XFBD and dexamethasone (DEX) could reduce the increase of the organ index caused by LPS (***P < 0.001 vs model) (Fig. 1C). In the representative photographs of spleen and lung tissue, the spleen in model group was swelling, lung had obvious edema, hyperemia, and congestion, meanwhile the XFBD and DEX could alleviate these symptoms (Fig. 1D). Severe congestion and enlarged red pulp were observed in the spleens of model mice. XFBD and DEX administration reduced the lung and spleen damage compared to the model group (Fig. 1E). Meanwhile, XFBD can significantly improve the lung function in mice by upregulating lung function results of tidal volume (TV), peak inspiratory flow (PIF), and minute volume (MV) indices (Fig. 1F). The local expression of pro-inflammatory IL-1β, IL-6, and TNF-α in serum was enhanced by LPS, while treatment with XFBD and DEX markedly reduced the levels of these three cytokines (***P < 0.001 vs model) (Fig. 1G). The experimental results suggested that XFBD at a dosage of 1.95 g/kg significantly and effectively mitigated the systemic inflammatory response induced by LPS in mice. Therefore, XFBD-L at this particular dosage (1.95 g/kg) was selected for subsequent experiments.

**Fig 2:**
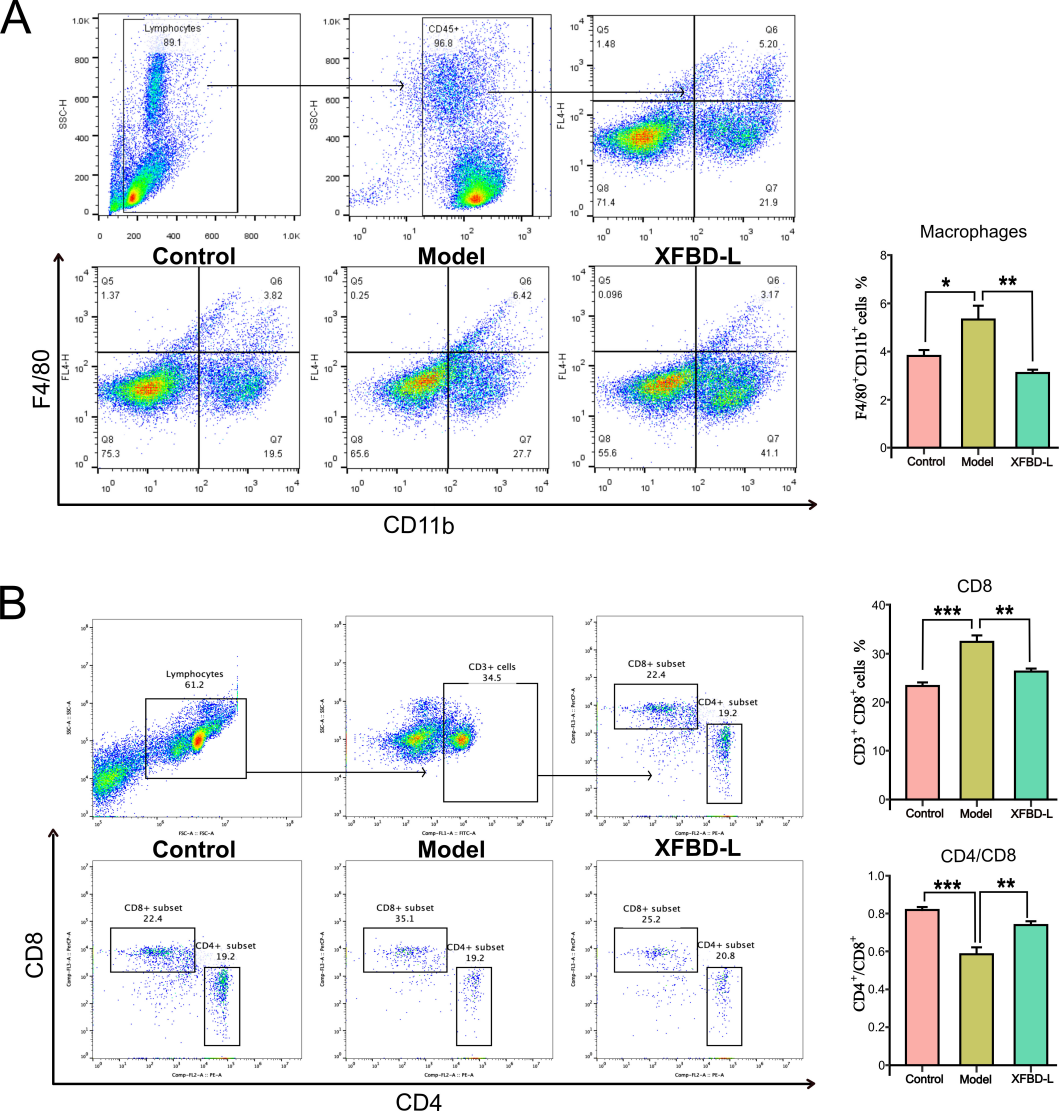
Flow cytometry was used to detect the proportions of macrophage and T cells in the spleen. (**A**) Flow cytometry analysis of the macrophage subsets in the spleen (*n* = 5 per group). (**B**) Flow cytometry analysis of the percentage of CD4^+^ and CD8^+^T cells in the spleen (*n* = 5 per group). Data were presented as mean ± SEM (*n* = 5 per group). Statistical significance was determined using one-way ANOVA, followed by Tukey test, ns means not significant, *P < 0.05, **P < 0.01, ***P < 0.001.

Immunity plays an important role in the progression and treatment of SIRS disease. As potent producers of cytokines, macrophages play a crucial role in regulating immune responses in SIRS. As shown in Fig. 2A, compared with the control group, the macrophage percentage was significantly increased in the LPS model group, while XFBD significantly downregulated the level of macrophage (**P < 0.01 vs model), suggesting that XFBD has a modulatory effect on innate immunity. Excessive cytokine could also be produced from activated T cells, which can contribute to the development of a cytokine storm, leading to severe inflammation and organ dysfunction. Our study demonstrated that XFBD reduces the LPS-induced increase in CD8^+^ T cell levels and decreases the CD4^+^/CD8^+^ ratio (**P < 0.01 vs model), thus exerting a regulatory effect on specific immunity (Fig. 2B). The above results indicated that 1.95 g/kg XFBD regulates macrophage and T-cell immunity, thereby regulating the immune response.

### XFBD modulates gut microbiome of LPS-induced SIRS mice

**Fig 3:**
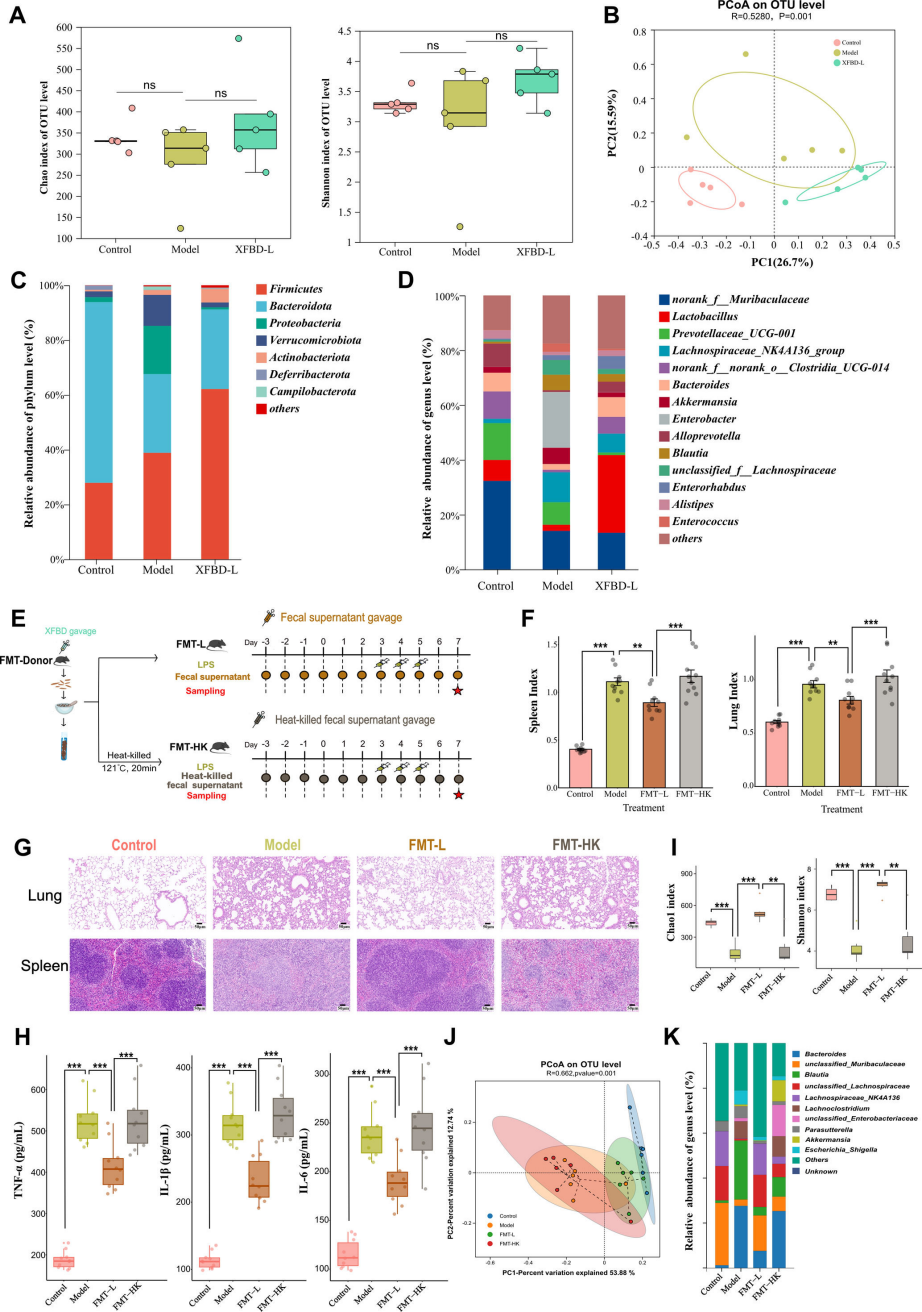
Comparative assessment of XFBD impact on the gut microbiome and subsequent fecal microbiota transplantation (FMT) effects on symptoms and microbiota in SIRS mice. (**A**) Alpha diversity analysis of different groups including Chao and Shannon indices. Data were presented as mean ± SEM (*n* = 5 per group). Statistical significance was determined using one-way ANOVA, followed by Tukey test, ns means not significant, *P < 0.05, **P < 0.01, ***P < 0.001. (**B**) The PCoA diagram is based on the bray curtis distances of different groups (*n* = 5 per group). (**C**) Bacterial abundance changes at phylum level of different groups (*n* = 5 per group).** (D**) Bacterial abundance changes at genus level of different groups (*n* = 5 per group). (**E**) Experimental scheme for fecal microbiota transplantation (FMT). XFBD-treated SIRS mice were used as donors, and two recipient groups of mice were subjected to daily gavage with either live or heat-killed fecal content for a period of 7 days. (**F**) Spleen and lung weight index of each group (*n* = 10 per group). (**G**) H&E staining of lung and spleen tissue sections (*n* = 6 per group). (**H**) The serum concentrations of TNF-α, IL-1β, and IL-6 in mice from different groups (*n* = 10 per group). (**I**) Alpha diversity analysis of different groups including Chao1 and Shannon indices (*n* = 6 per group). (**J**) The PCoA diagram is based on the weighted UniFrac distances of different groups (*n* = 6 per group). (**K**) Bacterial abundance changes at genus level of different groups. Data were presented as mean ± SEM (*n* = 6–10 per group). Statistical significance was determined using one-way ANOVA, followed by Tukey test. n.s. not significant, *P < 0.05, **P < 0.01, ***P < 0.001.

16S rRNA gene sequencing was used to analyze the gut microbiota diversity of each sample. As shown in Fig. 3A, although not statistically significant, there is a clear tendency for species abundance to decrease in the model group compared to the control group. XFBD treatment increased species abundance, suggesting that XFBD has the potential to counteract LPS-induced reductions in gut microbiome species complexity. Based on Bray-Curtis distances, principal coordinate analysis (PCoA) exhibited distinctly different clustering results of samples between the different groups (Fig. 3B), proving that XFBD consumption could cause alteration of microbiota community structure in mice. The changes in the bacterial community at the phylum level for each group were depicted in Fig. 3C. There was a tendency for the model group to exhibit a higher abundance of Proteobacteria, which was reduced following XFBD administration (Fig. S1A). Additionally, it was found that XFBD supplementation in SIRS mice led to the elevated proportion of *Lactobacillus* spp. as shown in Fig. 3D and Fig. S1B (*P* < 0.01 vs model). These findings collectively indicate that the administration of XFBD exerts a regulatory effect on the gut microbiota, promoting a shift toward a more balanced and beneficial microbial community in SIRS mice.

### XFBD-altered gut microbiota ameliorates the pathological symptoms in LPS-induced SIRS

The systemic inflammatory mouse model exhibits pronounced severity. Post gut microbiota clearance with antibiotics, a significant decrease in mouse survival is observed, the exact reason of which remains elusive. Notably, in certain studies, researchers have opted to utilize filtered (28) or autoclaved fecal material (29) as a control, a practice that seeks to elucidate and contextualize the intricate interplay between gut microbiota and inflammation. Therefore, we conducted FMT experiments in two separate SIRS mouse groups to validate the impact of XBFD-mediated microbiota. Fecal samples from the XFBD-fed group were administered via oral gavage to the first group, containing live microbiota (FMT-L), while the second group received fecal samples with heat-killed microbiota (HK-FMT) as the control group (Fig. 3E). The results demonstrated that FMT-L treatment led to a significant decrease in the relative weight of spleen and lung tissue (**P < 0.01 vs model), whereas FMT-HK treatment did not show such effects (Fig. 3F). Histological analyses of the lung and spleen tissues further confirmed that FMT-L treatment attenuated spleen indistinct red-white marrow demarcation and lung pathological changes, such as thickened alveolar septum, marked inflammatory cell infiltration, and increased lung parenchyma proportion (vs model), which were not observed in the FMT-HK groups (Fig. 3G). Additionally, FMT-L led to a reduction in the expression levels of TNF-α, IL-6, and IL-1β in the serum (Fig. 3H). Notably, the FMT-L group showed a noticeable increase in the Chao index compared to the model group, indicating enhanced gut microbial diversity. This trend was also observed in the Shannon index. On the other hand, the FMT-HK group did not exhibit a similar increase in gut microbial diversity (Fig. 3I). Based on weighted UniFrac distances, PCoA exhibited distinctly different clustering results of samples, demonstrating that FMT-L induces alterations in the microbiota community structure that are closer to the control group, while FMT-HK results in a composition more similar to the model group (Fig. 3J). At genus level, FMT-L treatment could remodel the gut microbiome after LPS induction, characterized by decreasing the abundance of *Escherichia-Shigella*, *LachnospiraceaeNK4A136*, *unclassified-Oscillospiraceae*, *Bacteroides*, and increasing the abundance of *Erysipelatoclostridium* and *Parabacteroides* (Fig. 3K; Fig. S2). Overall, FMT-L alleviated SIRS in mice more profoundly than FMT-HK and these effects may depend on interactions between XFBD administration and gut microbiotas.

### *In vitro* screening of GMRCs from XFBD

**Fig 4:**
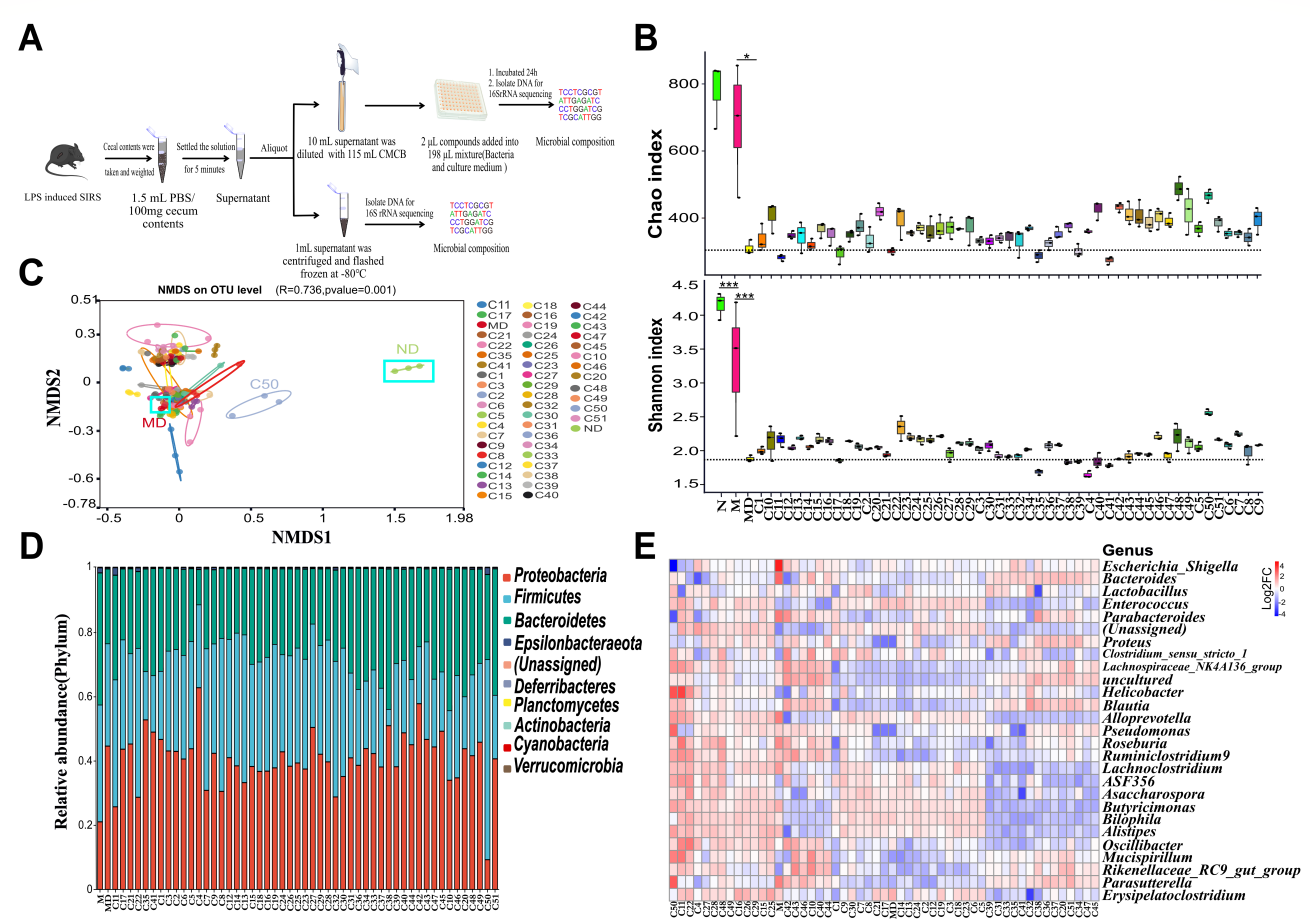
*In vitro* impact of XFBD-derived compounds on the composition of intestinal microbiome of SIRS mice.** (A**) Schematic overview of the screening approach and bioinformatics analysis. (**B**) Alpha diversity analysis of gut microbiota from C57BL/6 mice cecum cultured *in vitro* with various XFBD-derived compounds for 24 h. The dashed line indicates the richness of species in the control. Data were presented as mean ± SEM (*n* = 3 per group). Statistical significance was determined using one-way ANOVA, followed by Tukey test. *P < 0.05, **P < 0.01, ***P < 0.001. (**C**) NMDS analysis of genus abundance of LPS-induced SIRS in mice with XFBD-derived compounds treatment. The control group was shown in blue rectangle, ND stands for “cultured control,” MD stands for “cultured model.” The significance of community structure differences between groups is based on Bray-Curtis distance algorithm, distance difference matrix was calculated by Anosim analysis methods. (**D**) Bacterial taxonomic profiling at the phylum level. (**E**) Bacterial abundance changes at genus level caused by XFBD-derived compounds relative to an untreated control.

We used 51 individual compounds derived from 13 TCM herbs that constitute XFBD for the *in vitro* experiments. These compounds will be referred to as XFBD-derived compounds in the following context. Under strict anaerobic conditions, we collected fresh cecal content from mice with LPS-induced SIRS and administered with 0.9% NaCl (referred to as N). The collected cecal content was then thoroughly mixed with growth media and dispensed into 96-well plates (Fig. 4A). Each well was then supplemented with different XFBD-derived compounds and incubated anaerobically at 37°C for 24 h. To examine the effects of the culturing conditions on the gut microbiome, we analyzed the changes in the relative composition of the microbiota community from SIRS mice before and after *in vitro* culture, referred to as M and MD, respectively. The results showed that the culture process indeed influenced the microbial composition (Fig. S3). As shown in Table S4, the cultured model group (MD) maintained 62 bacterial taxa, a fairly representative number compared to the 80 taxa found in the uncultured model group (M). These results demonstrated that our culturing conditions retained sufficient bacterial diversity, enabling the *in vitro* assessment of relative changes in bacterial composition between GMRC-treated and cultured model group (MD).

The gut microbiota changes were analyzed using the Illumina MiSeq platform with paired primers targeting V3–V4 regions. A total of 3,008 OTUs were identified for the cecal microbiota, with an average of 1,040, 965, 623, and 380 OTUs for the N (un-cultured control), M (un-cultured model), ND (cultured control), and MD (cultured model) groups, respectively, at a 97% similarity level. The OTUs of the XFBD-derived compound treatment group ranged from 362 to 632 (Table S5). The rarefaction curve and rank abundance plot (Fig. S4A and B) indicated that the data covered both rare new phylotypes and the vast majority of the diversity.

To determine XFBD-derived compound concentrations for *in vitro* screening, we conducted minimum inhibitory concentration (MIC) assays against four representative laboratory strains of gut bacteria (Fig. S4C). Based on the MIC of XFBD-derived compounds, various concentrations were selected to test their impact on gut microbiota. After incubating for 24 h under anaerobic conditions, the change in OD_600_ was measured. Concentrations causing over 80% OD_600_ reduction were excluded due to adverse effects on mixed gut microbiota growth (Fig. S4D). Venn diagram of the composition of OTUs in cecal microbiota showed 114 common species (Fig. S4E).

Consistent with the *in vivo* experiments, the *in vitro* assay showed lower OTUs and species diversity (Chao and Shannon indices) in the M group (uncultured model) compared to the N group (uncultured control). Interestingly, most XFBD-derived compounds (C1–C51) increased the Chao and Shannon indices compared to the MD group (cultured model) (Fig. 4B). We then employed nonmetric multidimensional scaling (NMDS) using the Bray-Curtis dissimilarity at the OTU level, which revealed distinct gut microbiota compositions between different groups. Notably, some compounds, for example, C50, were closely aligned with the ND sample, indicating their potential to regulate the gut microbiome toward a healthy state (Fig. 4C). The ratio of Firmicutes to Bacteroidetes (F/B), the two main bacterial phyla in gut microbiota, is indicative of dysbiosis. The M group showed a low F/B ratio, while some compounds, including M50, M32, and M22, increased the F/B ratio (Fig. 4D; Fig. S4F). At the genus level, 27 most abundant genera were selected for further analysis, which showed that XFBD-derived compounds largely remodeling the gut microbiota in different patterns (Fig. 4E).

**Fig 5:**
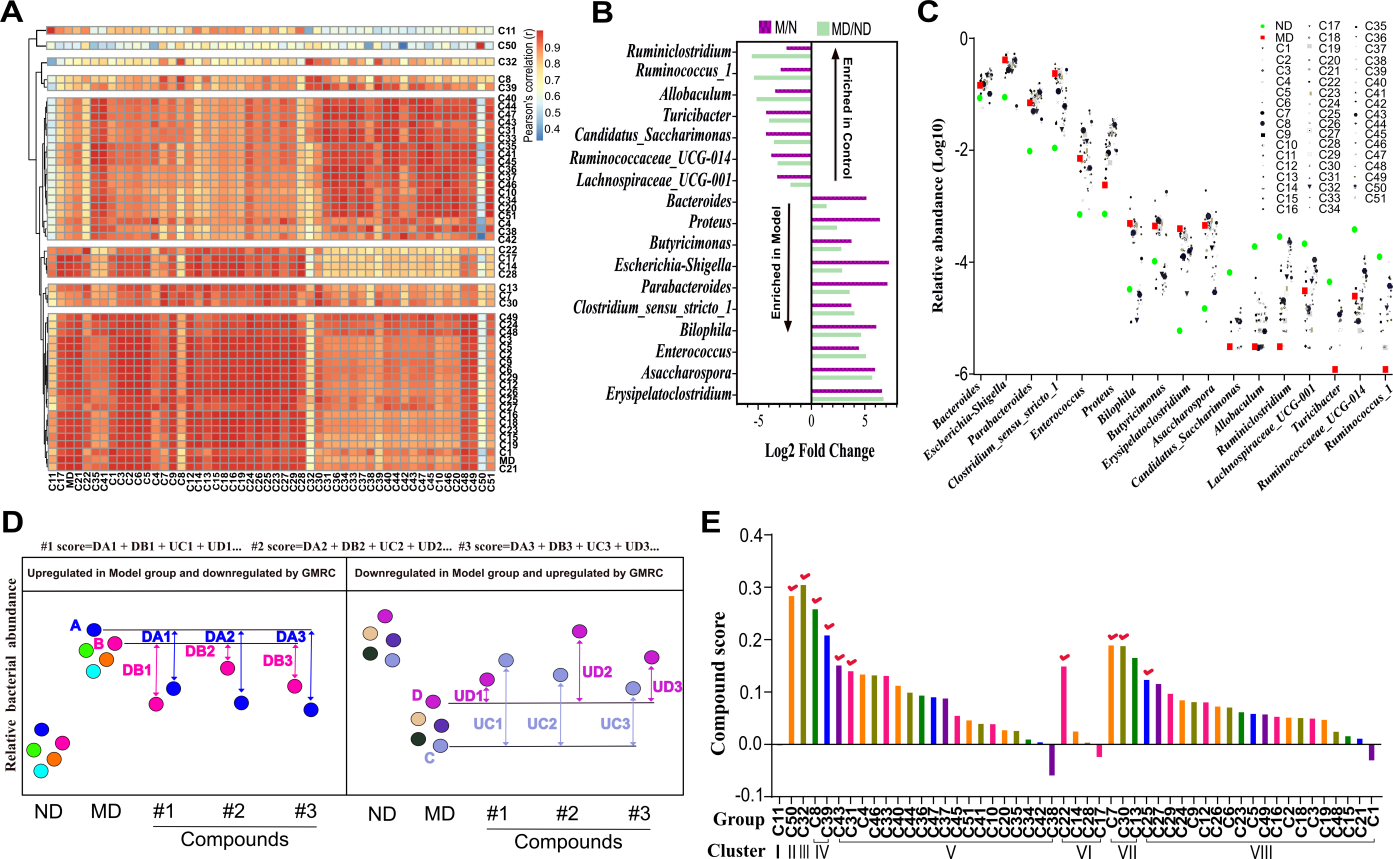
*In vitro* screening of gut microbiome remodeling compounds from XFBD formula. (**A**) Heat map showing pairwise comparison of different XFBD-derived compounds in remodeling mice gut microbiota, which is based on the total OTUs in compound treatment groups. Higher correlation indicates that those peptides show more similar effects in changing the microbiota composition. (**B**) A waterfall plot of significantly altered genus between model and control showing consistent trend in both uncultured and cultured groups. Differential abundance analysis was performed to assess variations in taxonomic abundances between groups using the negative binomial test (DESeq2, adjusted *P* < 0.05). (**C**) Scatter plot showing the specific seventeen genera changes after the XFBD-derived compounds treatment. The green and red dots, respectively, represent the control group and the model group. (**D**) Schematic diagram of distance-based scoring algorithm for XFBD-derived compounds. The algorithm considers not only the bacteria that increased in abundance, but also the bacteria that decreased in SIRS mice compared to control group (based on the 17 differential abundance generas). Compounds were scored by summing the observed changes in abundance for each of the identified bacteria, with shifts toward the ND state being considered positive and shifts away being negative. (**E**) The bar chart showing the scoring results of the XFBD-derived compounds based on the distance scoring algorithm. The potent GMRCs in each cluster are indicated with red tick. Data were presented as mean ± SEM (*n* = 3 per group). Statistical significance was determined using one-way ANOVA, followed by Tukey test. n.s. not significant, *P < 0.05, **P < 0.01, ***P < 0.001.

By conducting a thorough pairwise comparison of the OTU from different XFBD-derived compounds, we identified a total of eight distinct clusters, each exhibiting a unique impact on the gut microbiota (Fig. 5A). Seventeen genera showing similar trends between the uncultured model/control (M/N) and cultured model/control (MD/ND) were selected as key genera. These genera were used to quantitatively describe the effects of XFBD-derived compounds on the gut microbiome of SIRS mice through differential abundance analysis with DESeq2 (Fig. 5B). The results revealed that compound treatments led to different outcomes for various genera, either pushing the genus away from the healthy state (ND) or toward it (Fig. 5C). To quantify the regulatory effects of each compound on the gut microbiota, a distance-based scoring algorithm was carried out as shown in Fig. 5D. The algorithm calculates the absolute value of the distance that a particular compound shifted each key genus closer to the ND group compared to the MD group, and then summed these values. This reflects how closely each compound can regulate the gut microbiome toward a healthy state (Fig. 5E). Based on these scores, we identified the top-performing compounds within each of the eight clusters. These high-scoring compounds were then combined to formulate GMRC cocktails.

### Designing of GMRC cocktails simplified from XFBD

By leveraging the power of *in vitro* high throughput screening, we were able to identify and cluster potential GMRCs with similar gut microbiota regulating characteristics. Based on the quantitative evaluation of the gut microbiota regulation ability of XFBD-derived mono-compounds, we developed various GMRCs cocktails simplified from XFBD by selecting representative GMRCs from each cluster (Table S2). The idea was to take one representative from each cluster to create cocktails that (partially) restore the gut microbiome regulation and therapeutic activities of the XFBD formula. Subsequently, we conducted *in vitro* evaluations to investigate the remodeling effects of different cocktails on the gut microbiota.

**Fig 6:**
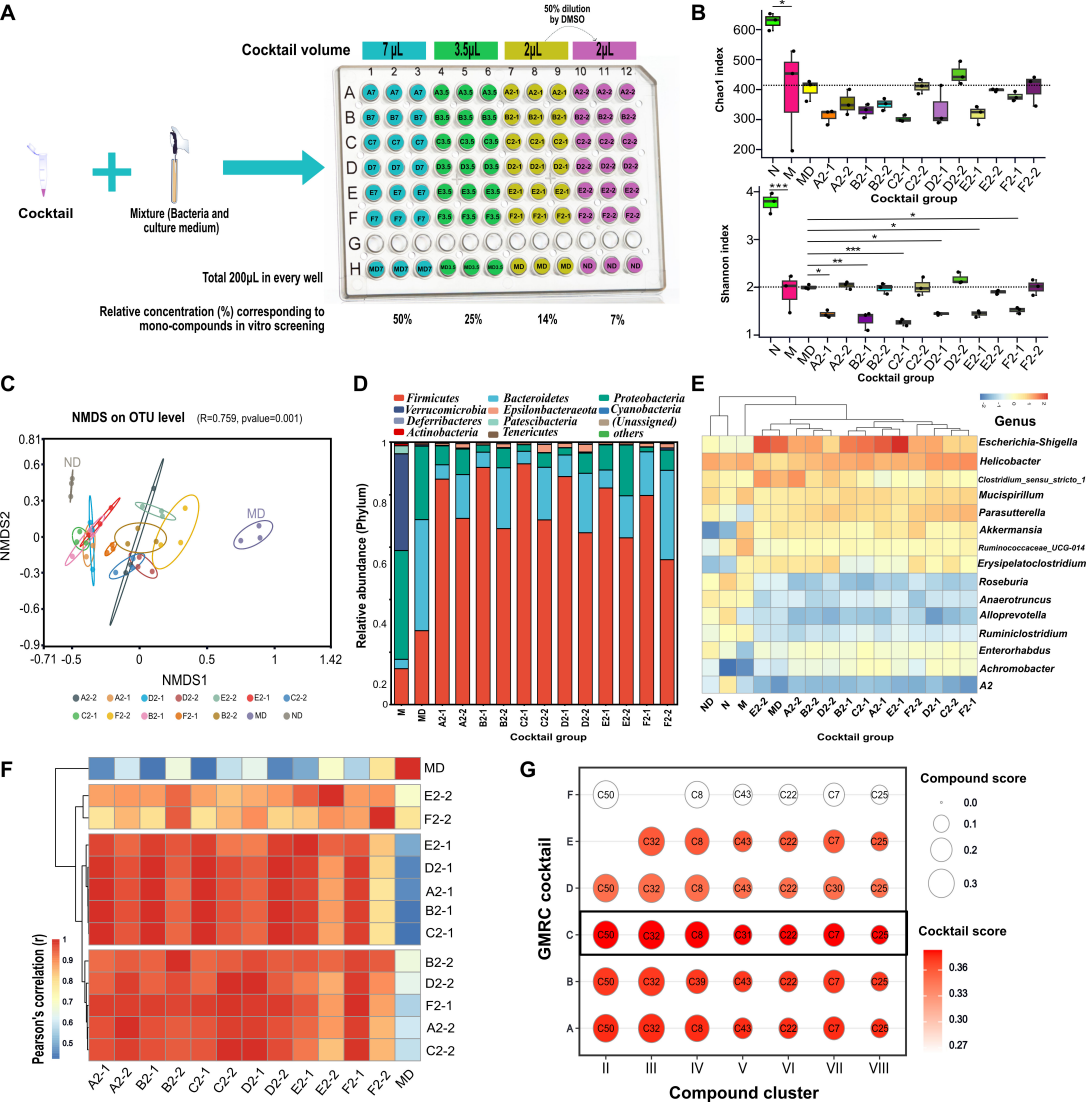
*In vitro* evaluation the gut microbiome remodeling activity of GMRC cocktails simplified from XFBD formula. (**A**) Diagram illustrating the design of GMRC cocktails concentration. (**B**) Alpha diversity analysis of gut microbiota from SIRS mice cecum cultured *in vitro* with various GMRC cocktails for 24 h. The dashed line indicates the richness of species in the control. Data were presented as mean ± SEM (*n* = 3 per group). Statistical significance was determined using one-way ANOVA, followed by Tukey test. n.s. not significant, *P < 0.05, **P < 0.01, ***P < 0.001. (**C**) NMDS analysis of genus abundance of LPS-induced SIRS in mice with GMRC cocktail treatment based on the weighted UniFrac distances. (**D**) Bacterial taxonomic profiling at the phylum level caused by GMRC cocktails. (**E**) Differential abundance bacterial changes at genus level, linear discriminant analysis effect size (LEfSe) analyses between SIRS mice and control mice (M vs N, MD vs ND). (**F**) Heat map showing pairwise comparison of different GMRC cocktails in remodeling mice gut microbiota. (**G**) The bubble chart displays the scoring results of the GMRC cocktails based on the distance scoring algorithm. The numbers in the bubbles indicate the number of compounds.

To determine the optimal concentration for GMRC cocktails, we mixed the DMSO stock solution of each mono-compound in equal ratios. We selected one representative compound from each of the eight clusters shown in Fig. 5A, except for C11 from cluster I due to its very low score. Starting with 1 µL of each proposed cocktail component, we prepared a mixed stock solution. We then added 7, 3.5, and 2 µL of this mixed stock solution to the incubation medium containing cecum microbiome, reaching a final volume of 200 µL (Fig. 6A). As depicted in Fig. S5A and B, the addition of 7 and 3.5 µL of the mixed stock solution resulted in a drastic decrease in alpha diversity and was therefore excluded from further consideration. Figure 6B indicated that GMRC cocktail concentration significantly affected its regulatory ability. Low concentrations have minimal impact on gut microbiota diversity in SIRS mice, while high concentrations reduce alpha diversity. Additionally, the NMDS analysis at the OTU level revealed that cocktail C showed the closest resemblance to the control (ND) treatment (Fig. 6C). Almost all cocktail treatments significantly increased the abundance of Firmicutes, thereby improving the F/B ratio (Fig. 6D; Fig. S5C).

At the genera level, we observed a significant increase in the abundance of *Bacteroides*, *Escherichia Shigella*, *Enterobacte*r, and *Enterococcus* in the cultured cecum from model mice (Fig. 6E; Fig. S5D). However, cocktail combinations A, B, C, D, and E were able to mitigate this increase to some extent (Fig. 6E; Fig. S5D). As shown in Fig. 6F, the gut microbiota regulation profiles of cocktail combinations A, B, C, and D were highly similar, as they clustered together. This finding suggests that compounds 50 and 32 may serve as key factors in the regulatory function of XFBD. Moreover, the distance-based scoring algorithm demonstrated that cocktail C had the highest score in terms of gut microbiota regulation, transitioning from a state of SIRS to a normal state (Fig. 6G). These results emphasize the potential significance of cocktail C in modulating the gut microbiota toward a healthier and balanced state, suggesting its potential as a promising therapeutic intervention for addressing SIRS and restoring gut microbial homeostasis.

### Therapeutic effects of selected GMRC cocktail on SIRS mice

**Fig 7:**
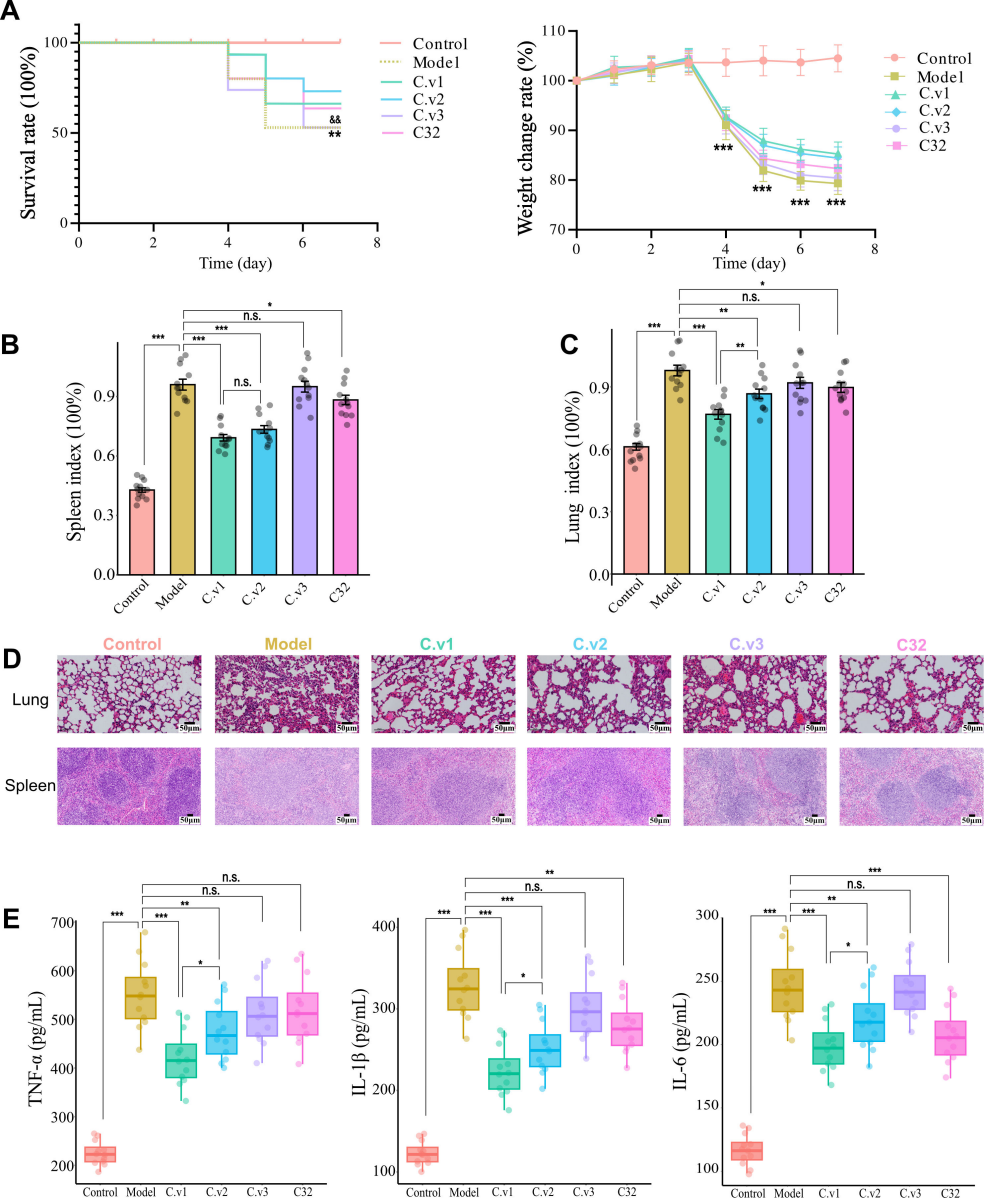
Theraputic effects of selected GMRC-cocktails on SIRS mice. (A) The survival rate and the body weight change of different groups of mice (n=12 per group). Statistical signifcance was determined using two-way ANOVA, followed by Tukey test. *P ≤ 0.05, **P ≤ 0.01, ***P ≤ 0.001 was model relative to control group, ^&^*P* ≤ 0.05, ^&&^*P* ≤ 0.01, ^&&&^*P* ≤ 0.001 was compound 32 relative to model group. (B) Splenic index (*n* = 12 per group). (C) Lung index (*n* = 12 per group). (D) H&E staining of lung and spleen tissue sections (*n* = 6 per group). (E) The serum concentrations of TNF-α, IL-1β, and IL-6 in mice from different groups (*n* = 12 per group). Data were presented as mean ± SEM (*n* = 12 per group). Statistical signifcance was determined using one-way ANOVA, followed by Tukey test. n.s. not significant, *P < 0.05, **P < 0.01, ***P < 0.001.

To explore the therapeutic effects of cocktail C and the individual contributions of its mono-compounds, we devised different compound combinations for evaluation. These combinations were named C.v1 (aucubin, gentiopicroside, syringic acid, gallic acid, p-Hydroxybenzaldehyde, para-hydroxybenzoic acid, and isoimperatorin), C.v2 (aucubin, syringic acid, gallic acid, p-hydroxybenzaldehyde, para-hydroxybenzoic acid, and isoimperatorin), C.v3 (syringic acid, gallic acid, p-hydroxybenzaldehyde, para-hydroxybenzoic acid, and isoimperatorin), and compound 32 (gentiopicroside). These combinations were then evaluated for their effects on SIRS mice. The results demonstrated that the administration of C.v1, C.v2, and compound 32 exerted a remarkable impact on reducing mortality and mitigating weight loss in mice with SIRS (Fig. 7A). The severity of spleen and lung injury was also analyzed through detecting spleen index, lung index, and histological examination. Our results revealed a significant reduction in spleen and lung indices in both the C.v1 and C.v2 groups compared to the model group. Notably, C.v1 exhibited more pronounced effects in attenuating the indices of spleen and lung (Fig. 7B and C). These results were confirmed by a histological examination of H&E stained lung and spleen tissue sections (Fig. 7D). Mice in the model group showed congestion of lung bronchi, obvious destruction of the alveolar septum, and inflammatory cell infiltration in the interspace. In contrast, C.v1, C.v2, and compound 32 administration resulted in less congestion of bronchi, clear alveolar septum structures, and decreased infiltration of inflammatory cells. Consistently, extensive hemorrhage was identified in the spleens of mice in the model group, with obvious atrophy of cortical lymphoid nodules, dilation of the medullary area, congestion of splenic sinuses, and increased inflammatory cell infiltration. However, mice in the C.v1, C.v2, and compound 32 groups successfully eliminated these pathological manifestations. Moreover, the model group exhibited a significant increase in the levels of proinflammatory cytokines TNF-α, IL-1, and IL-6. Substantial reductions in the elevated cytokine concentrations were observed upon treatment with C.v1 and C.v2. Compound 32 also lowered cytokine levels, although to a lesser extent than C.v1 and C.v2 (Fig. 7E). Notably, the therapeutic effects of C.v3 were not evident in our study. These results strongly suggest that GMRC cocktail C holds promise as a potential therapeutic intervention for managing SIRS and alleviating its inflammatory effects. The observed therapeutic effects point to the keystone roles played by both compound 50 and compound 32 within the cocktail.

### *In vitro* effects of the GMRC cocktail on fecal microbiota of SIRS patients

**Fig 8:**
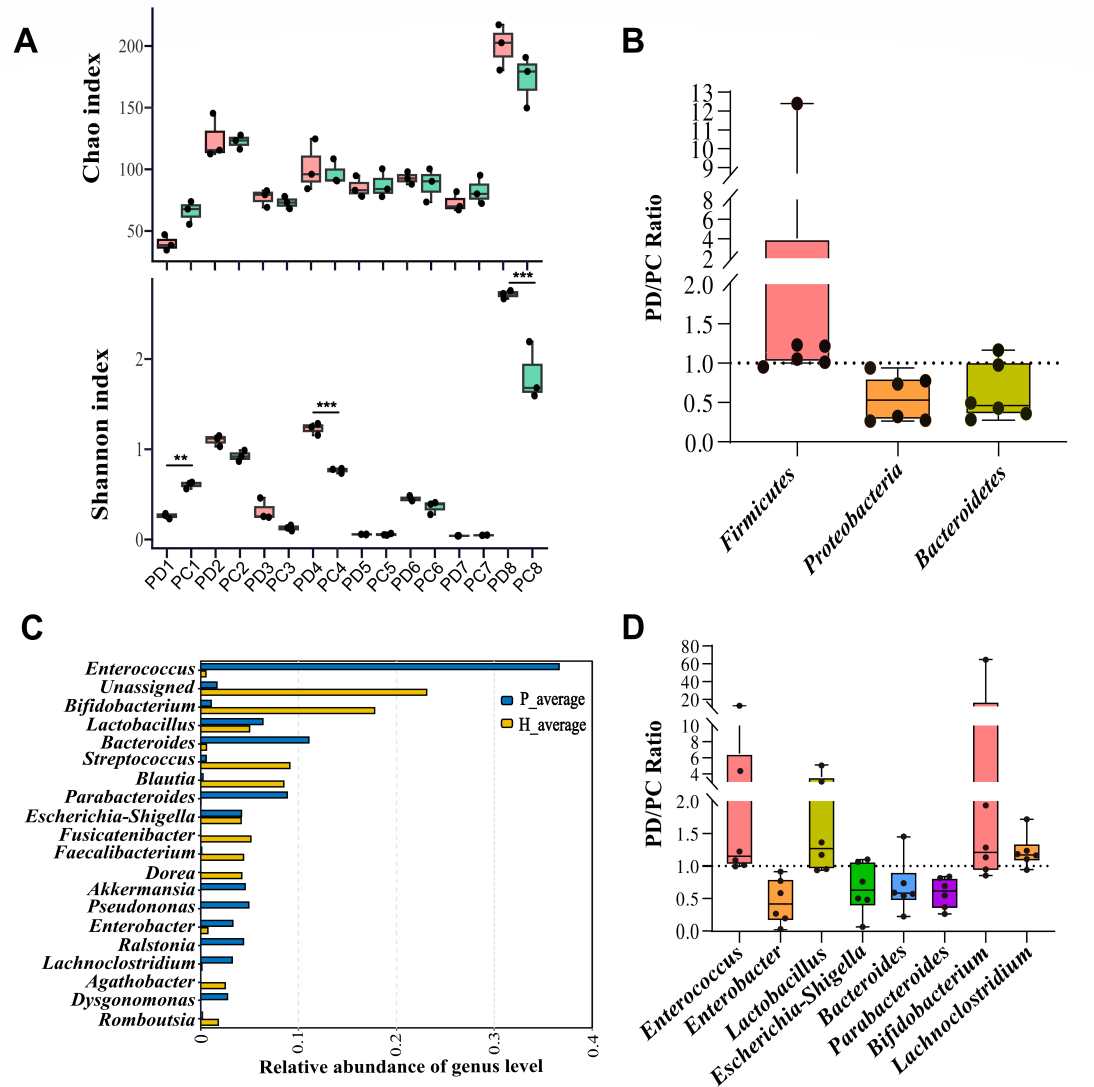
Regulatory effects of GMRC cocktail C on fecal microbiota of SIRS patients. (**A**) Alpha diversity analysis of gut microbiota from SIRS patients cultured *in vitro* with GMRC cocktail C for 24 h. PC: samples treated with cocktail C; PD: samples with DMSO as a control. (**B**) Specific changes in phylum level for each individual patient after GMRC cocktail C treatment. (**C**) Abundance comparison of key genera between SIRS patients and healthy volunteers. (**D**) Specific changes in genus level for each individual patient after GMRC cocktail C treatment.

The fecal microbiota of eight SIRS patients was examined during a 24-hour incubation with cocktail C using the *in vitro* culture system. The treatment with GMRC cocktail C did not significantly alter the alpha diversity of most fecal samples, except for sample 1, which showed an increase in both Chao1 and Shannon indices, and sample 8, where a slight decrease was observed in both indexes (Fig. 8A). At phylum level, the abundance of Firmicutes increased while Bacteroidetes and Proteobacteria decreased, resulting an increased F/B ratio (Fig. 8B; Fig. S6A and B).We also sequenced fecal samples from ten healthy volunteers and compared their microbial composition with that of SIRS patients (Fig. 8). Our results are consistent with previous studies, which reported that SIRS patients had increased levels of *Enterobacter, Escherichia Shigella*, *Bacteroides*, and *Parabacteroides*, along with decreased levels of *Lactobacillus* and *Bifidobacterium* (30–32). Following treatment with cocktail C, the levels of *Enterobacter*, *Escherichia-Shigella*, *Bacteroides*, and *Parabacteroides* decreased, while the levels of *Enterococcus*, *Lactobacillus*, and *Bifidobacterium* increased (Fig. 8D; Fig. S6C).

## DISCUSSION

The manipulation of gut microbiota has been shown to have a significant impact on human health, with emerging evidence pointing to the possibility of using microbiota-based interventions to prevent and treat various diseases (33–35). SIRS remains a significant global cause of mortality (36). TCM has showed potential in reducing mortality among septic patients. Notably, Xuebijing injection (XBJ), an herbal-based intravenous preparation, significantly reduced 28-day mortality in sepsis patients compared to the placebo in a randomized clinical trial (37). Recently, attention has shifted toward investigating the role of the gut microbiome and its potential contribution to SIRS, making it a subject of increasing interest in research (38). In this regard, the use of small molecules as GMRCs has garnered attention as a promising strategy to address the limitations of current manipulation methods, such as FMT, probiotics, and prebiotics interventions. Compared to live biotherapeutics, small molecules offer the advantage of increased targeting specificity, potentially enabling oral delivery in the form of pills without reducing activity. Moreover, small GMRCs offer firms various practical benefits including simple approval and well-established manufacturing and distribution (20). While there has been significant progress in the development of small molecules targeting the gut microbiome, the current pool of compounds capable of precisely regulating the microbiota remains limited.

Traditional Chinese medicine utilizes multi-herbal formulations for the treatment of various diseases. Recent studies have demonstrated that certain multi-herbal formulations possess the ability to modulate the gut microbiota, leading to improved metabolic health and reduced inflammation in specific diseases (39). These formulations present a unique advantage for the discovery of GMRCs, as they contain complex mixtures of herbs that may have synergistic effects not found in single compounds (40). Therefore, the discovery of GMRCs from complex TCM prescriptions is of significant importance. In recent years, researchers have discovered active compounds in some TCMs that exert therapeutic effects by modulating the gut microbiota (41). Studies have revealed that natural polysaccharides are among the most potent modulators of gut microbiota composition and can benefit human health (42). Berberine, an alkaloid found in herbal plants like *Berberis vulgaris* and *Coptis chinensis*, can treat various diseases such as cancer, inflammation, bacterial infections, and fatty liver disease. Animal model studies have disclosed that BBR can ameliorate diseases by influencing the gut microbiota composition (43). The TCMs repertoire encompasses a wide array of bioactive compounds, such as flavonoids (e.g., baicalin) (44), saponins (e.g., ginsenoside) (45), and polyphenols (e.g., curcuminoid) (46) that have significant modulatory effects on the gut microbiota.

Most research to date has utilized mice as model organisms to study how compounds influence the assembly of microbial communities (47). The traditional trial-and-error approach in animal studies often proves ineffective for quantitatively comparing the effects of a large number of GMRCs in a single batch. This study employed an *in vitro* model to achieve high-throughput screening of 51 GMRCs derived from XFBD formula, for their potential to regulate the gut microbiome of SIRS mice. By using this *in vitro* approach, we were able to score and cluster all the compounds based on their gut microbiome regulation effects. This method allowed for a more efficient and systematic evaluation compared to conventional animal studies. However, despite our efforts to optimize the culture conditions, it is important to mention the limitations of our *in vitro* culture process. Specifically, our method reduced the diversity of the gut microbiome to some extent. Nevertheless, our *in vitro* conditions preserved 62 bacterial taxa, which is fairly representative compared to the 80 taxa found in the uncultured community (Table S4). Emerging technologies, such as “intestine-on-a-chip” systems that allow for the coculture of living human intestinal epithelium, hold promise for further improving the preservation of bacterial diversity. These advanced models are better suited to sustain obligate anaerobic bacteria, potentially increasing the number of bacterial taxa (48, 49). Incorporating such technologies in future studies could further improve the efficiency and accuracy of drugs targeting the gut microbiome.

*In vitro* evaluation of compounds on microbiota has been applied to assess the impact of non-antibiotic drugs on human gut bacteria (21). Chen et al. employed an *in vitro* microbiota culturing approach to conduct a large-scale screening of cyclic D,L-α-peptides targeting the gut microbiota for potential anti-atherosclerosis activity (22). In this study, we were able to quantitatively evaluate the potential of 51 compounds in regulating dysbiosis microbiota to healthy state. The results revealed that compounds 50 (aucubin), 32 (gentiopicroside), 8 (syringic acid), 39 (liquiritigenin), and 22 (p-hydroxybenzaldehyde) were particularly effective in remodeling the gut microbiota in the *in vitro* experiments (Fig. 5I). Indeed, liquiritigenin and p-hydroxybenzaldehyde have previously been associated with anti-inflammatory effects through their ability to modulate the gut microbiota (50–52). This reinforces the robustness and effectiveness of our *in vitro* screening approach, showcasing its potential in identifying promising compounds with anti-inflammatory properties based on their interactions with the gut microbiota.

In this study, we have designed a GMRC cocktail consisting of seven representative compounds that exhibited more potent therapeutic effects against SIRS compared to individual compounds (Fig. 6). Previous reports have indicated that multi-strain probiotic cocktails are more effective in regulating the gut microbiota against various illnesses than single strains (53, 54). Additionally, the administration of phage cocktails has proven to be successful in inhibiting pathobionts responsible for non-communicable diseases (55, 56). A bacteriophage cocktail has been developed to improve the growth performance and gut microbiome of broiler chickens (57). Interestingly, a mixture of chemically diverse synthetic glycans has shown remarkable effectiveness in altering the microbiome to promote health, surpassing the effects of using pullulan or galacto-oligosaccharides (58). Despite several studies reporting on the therapeutic effects of mono-GMRC, there hasn't been any research on the GMRC cocktail itself. This study has opened new possibilities for the development of gut microbiota-targeting cocktails, showing promising potential for future applications.

TCM are known for their complex chemical composition due to the combination of multiple herbs. Understanding the specific chemical constituents responsible for the therapeutic actions of TCM is a critical aspect of advancing their clinical applications and optimizing their therapeutic benefits (59). In this context, this study focuses on the gut microbiota as a target to identify an optimal cocktail simplified from the XFBD formula, which has shown significant potential in managing SIRS in mice and restoring gut microbiome homeostasis in SIRS patients (Fig. 7). Although this study sheds light on the potential of XFBD-derived GMRC cocktail in treating SIRS and regulating gut microbiome, it also highlights the need for further research. Given the limited understanding of the complete composition of XFBD and the availability of XFBD-derived compounds, our study utilized a set of 51 representative compounds for initial screening. However, it is worth noting that a more extensive pool of compounds, with specific concentrations of each compound, could potentially lead to the design of more effective cocktails with enhanced therapeutic potential. Moreover, further comprehensive studies and technological advances are essential to deepen our understanding of XFBD, the selected GMRC cocktails, and their mechanisms of action. Additionally, investigating potential adverse effects and drug interactions is essential to ensure their safe and effective use in clinical settings.

## MATERIALS AND METHODS

The XFBD formula was obtained from the State Key Laboratory of Component-based Chinese Medicine at Tianjin University of Traditional Chinese Medicine. All the ingredients in XFBD were of analytical grade and procured from Shanghai Yuanye Biotechnology Co. Ltd. These compounds were dissolved in DMSO for subsequent use. More detailed information about the compounds can be found in Table S1. Additionally, LPS was purchased from Sigma (L2630, St. Louis, MO, USA), and the TNF-α, IL-6, and IL-1β enzyme-linked immunosorbent assay (ELISA) kits were purchased from Sinobestbio (YX-091206M, Shanghai, China).

### Animal model

Six-week-old C57BL/6 specific pathogen-free male mice (18–22 g) were obtained from Beijing Vital River Laboratory Animal Technology Co., Ltd. (Beijing, China), with 12 mice per group (*n* = 12). The mice were housed in standard polycarbonate cages with four mice per cage, under controlled environmental conditions of 23 ± 2°C, humidity of 45% ± 10%, and 12-h light-dark cycles. They were provided with a standard diet and had free access to water throughout the study. The bedding among groups was mixed and updated frequently to minimize the individual variability and cage differences of microbiota among mice. All animal experiments were conducted in accordance with the Guide for the Care and Use of Laboratory Animals: Eighth Edition. The experimental procedures were performed following the guidelines approved by the Institutional Animal Care and Use Committee of Northwest A&F University and the Institutional Animal Care and Use Committee of Tianjin University of Traditional Chinese Medicine the under protocol number XN2023-0721 and TCM-LAEC2022191.

The mice were randomly divided into five groups: (i) the control group, (ii) the SIRS model group (LPS), (iii) the DEX group (LPS + 5 mg/kg Dexamethasone), (iv) the XFBD-L group (LPS + 1.95 g/kg XFBD formula), and (v) the XFBD-H group (LPS + 3.9 g/kg XFBD formula). The LPS induced SIRS mice model was established following previous studies with appropriate modifications (60). After a 1-week acclimatization period, during which the mice were monitored for health and stability, those exhibiting no obvious abnormalities were selected, marking this day as day 0 of the experiment. The model, DEX, XFBD-L, and XFBD-H groups received LPS (5 mg/kg) injections in the tail vein on days 3 to 5. Simultaneously, the control group was administered with 0.9% NaCl (27). XFBD and DEX groups received XFBD formula or dexamethasone administrations via continuous gavage from day 0 to day 7, while the model group received the same volume of double distilled water (Fig. 1A). Subsequently, all the mice were euthanized under deeply anesthetized conditions after undergoing morphology examinations and pulmonary function assessment using the pulmonary system (61) (IOX2 Software, EMKA Technologies, France).

### Fecal microbiota transplantation

Six-week-old C57BL/6 specific pathogen-free male mice (18–22 g) were obtained from Beijing Vital River Laboratory Animal Technology Co., Ltd. (Beijing, China), with 12 mice per group (*n* = 12). To prepare for the FMT procedure, fresh feces were aseptically collected from LPS-induced SIRS mice treated with XFBD (1.95 g/kg). Fresh feces samples were obtained from the donor group and combined to form a pooled mixture. The mixture was then homogenized and diluted in sterile saline, resulting in a final concentration of 100 mg of feces per milliliter. After thorough mixing, the suspension was allowed to settle, and a 200-µL aliquot of the supernatant was collected. This collected supernatant served as the transplant material within the subsequent 7-day period. The fecal supernatant was prepared 10 min prior to each FMT session to ensure minimal disturbance in bacterial populations during the procedure. Two groups of recipient mice were then administered a daily oral gavage of 100 µL/g fresh transplant materials, one group receiving heat-killed materials and the other serving as a control (heated to 121°C for 20 min), for a consecutive 7-day period.

### Tissues and samples

The mice were anesthetized with tribromoethanol, and blood samples were collected from the retroorbital sinus using 1.5 mL tubes. The collected blood was allowed to settle and then centrifuged at 3,000 × *g* for 15 min at 4°C. The resulting supernatant was stored at −80°C for future use. Subsequently, the mice were euthanized by spinal dislocation between 09:00 and 12:00 a.m. The lung and spleen tissues were harvested and weighed. The lung and spleen indices were calculated using the following formula: organ index  =  mean wt of organ (g)/body weight (g) ×  100% (62, 63). These tissues were fixed in 4% paraformaldehyde and embedded in paraffin. Five-micrometer-thick tissue sections were then deparaffinized and rehydrated using a xylene-ethanol-water gradient system. The sections were stained with H&E for light microscopic examination using an optical microscope (Nikon, Japan). Fecal samples were collected fresh at 7 days of treatment and immediately stored in frozen condition. All samples were stored at −80°C until analysis.

### Cytokines and flow cytometry

Serum concentrations of IL-6, IL-1β, and TNF-α were quantified using ELISA kits, following the manufacturer’s instructions (Sinobestbio, Shanghai, China). For the characterization of T cell and macrophage levels in mouse spleen, flow cytometry was employed according to the previous study (25). Single cells were isolated from the spleen and stained with fluorochrome-conjugated antibodies against the CD3, CD4, CD8, CD45, CD11b, and F4/80 cell surface proteins. After staining, the cells were fixed overnight with fixation solution (421002, BioLegend, USA) for detection. Images of all samples were acquired using the BD Accuri C6 Plus Flow Cytometry system (BD Biosciences, USA).

### 16S rRNA gene sequencing and data analysis

Fecal samples from the *in vivo* experiment and the cecal samples from the *in vitro* experiment were collected fresh at the last days of treatment , immediately frozen in liquid nitrogen, and stored at −80°C until analysis. Microbial genomic DNA was extracted using the PowerSoil DNA isolation kit (MoBio Laboratories Inc., Carlsbad, CA, USA). The 16S rRNA region was amplified by PCR using primers targeting the V3–V4 regions (5′-CCTACGGGAGGCAGCAG-3′, 5′-GGACTACHVGGGTWTCTAAT-3′) and then sequenced using an Illumina MiSeq platform with paired primers. Sequencing libraries were prepared using the NEBNext Ultra II DNA Library Prep Kit for Illumina (New England Biolabs, MA, USA) following the manufacturer’s recommendations, and index codes were added. The library’s quality was assessed using the Qubit 2.0 Fluorometer (Thermo Fisher Scientific, MA, USA). Finally, the library was sequenced and 250 bp paired-end reads were generated using the Illumina NovaSeq platform.

The quality of the raw data was assessed using Fastp version 0.14.1, utilizing a sliding window approach with parameters -W 4 and -M 20 to trim any windows with an average quality score below 20. The primers were eliminated by using cutadapt software (https://github.com/marcelm/cutadapt/) to obtain the paired-end clean reads. The UPARSE method was applied to define operational taxonomic units (OTUs) (64).

### MIC of XFBD-derived compounds against selected bacterial strains

The MIC assay was conducted using bacterial strains, including *Clostridium clostridiiforme* TS340077, *Bacteroides fragilis* ATCC 25285, *Bifidobacterium bifidum* ATCC 29521, and *Proteus mirabilis* CMCC 49005. The selection of these strains was based on their belonging to the two major bacterial phyla (Firmicutes and Bacteroidetes) found in the gut, or being well-known probiotics (*B. bifidum*) from the Actinobacteria phylum. Culturing of the strains was carried out in a vinyl anaerobic chamber, using the media suggested by the supplier. The XFBD-derived compounds were individually dissolved in DMSO at varying concentrations and then stored in small aliquots at −20°C after freezing. The MIC assay was carried out using 96-well plates inside an anaerobic chamber. Bacterial strains were inoculated into 96-well plates with an initial OD_600_ of 0.05. Varying concentrations of XFBD-derived compounds were added, and the microplate was incubated at 37°C for 24 h. Bacterial growth was then assessed using a microplate reader (Tecan Spark, Tecan, Switzerland). The MIC of the tested compounds against the bacterial strains was defined as the lowest concentration inhibiting over 90% of bacterial growth.

### *In vitro* cecum microbiota culturing and GMRC screening

The cecum from mice was collected and all assays were carried out in an anaerobic environment using a laboratory anaerobic chamber (Defendeor AMW1000, Horiolab, Guangdong, China), as previously described (22). To enhance the reliability of the results and minimize the potential differences between individual mice, the experimental assay was conducted by pooling together the cecum contents obtained from three co-housed mice (Fig. 5A). The cecum contents were suspended in phosphate-buffered saline (PBS) (1.5 mL PBS per 100 mg cecum content) and left to settle for 5 min. The upper phase was then collected. From this, a 1.0-mL aliquot of the supernatant was centrifuged (8,000 × *g* for 3 min) to obtain the bacterial pellet, which was flash-frozen at −80°C for use as the uncultured control. Another 10 mL portion of the upper phase from the settled cecum suspension was diluted with 115 mL of Chopped Meat Carbohydrate Broth (Solarbao Technology Co., Ltd., China), resulting in a total volume of 125 mL of inoculated medium for *in vitro* GMRC screening. A combination of 198 µL of the inoculated medium and 2 µL of a stock solution containing an XFBD-derived compound was incubated for 24 h at 37°C in a 96-well plate. After measuring the absorbance, the bacterial solution was immediately sucked out and subjected to centrifugation. The resulting cell pellet was flash-frozen, and 16S rRNA sequencing was performed to analyze the microbial composition change of each well.

### Therapeutic effects of GMRC cocktail on SIRS mice

The *in vitro* quantitative study has indicated that cocktail C exhibited the most promising effects in terms of regulating the gut microbiota of SIRS mice. Based on the *in vitro* assay, our subsequent investigation aimed to determine whether promising compounds in the cocktail are essential and whether the cocktail itself is superior to individual compounds when treating SIRS mice. The mice were subjected to the same procedures as previously described and were randomly divided into seven groups for the study: (i) the control group (NC), (ii) the LPS group (model), (iii) the Com-1 group, (iv) the Com-2 group, (v) the Com-3 group, and (vi) the compound 32 group. The doses of the compounds were determined through preliminary trials and literature data (65, 66), and their specific quantities can be found in Table S2. The treatment and sampling procedures were carried out as explained in Fig. 5A. After sacrificing the mice, the collected samples were analyzed accordingly.

### *In vitro* effects of GMRCs on fecal microbiota of SIRS patients

The study protocol was approved by the Ethics Institutional Review Board of Tang Du Hospital, Fourth Military Medical University (permit no. K202301-09). Symptomatic, gender, and body mass index of volunteers are listed in Table S3. SIRS symptom was determined using Sepsis-3 criteria. In compliance with the principles of the Declaration of Helsinki and under the supervision of the ethics committee of Tang Du Hospital of Fourth Military Medical University. Fecal samples (200–300 mg) were collected from the eight SIRS volunteers in sterile plastic containers and put it in an anaerobic bag immediately. The collected samples were subsequently transferred to a laboratory anaerobic chamber and mixed with PBS at a ratio of 1.5 mL of PBS per 100 mg of feces sample. The resulting upper phase was further diluted with CMCB at a ratio of 1:11.5 (vol/vol). Following this, 2 µL of GMRC cocktail-C was added to 198 µL of the suspension. After incubating for 24 h, samples were collected and subjected to 16S rRNA sequencing analysis.

### Statistical analysis

All data were presented as the mean  ±  standard error of means (SEM) of at least three independent experiments for a given sample. For microbiota diversity, Chao and Shannon indices were carried out using vegan packages of R tools. To evaluate the difference in heterogeneous community structure between groups, principal coordinate analysis was performed based on weighted Unifrac distances of Bray-Curtis dissimilarity using the vegan package, followed by adonis analysis. Pearson’s correlation coefficient was computed to analyze the composition of microbiome after treatment with different compounds using the stats package. Differential abundance analysis was performed to assess variations in taxonomic abundances between groups using the negative binomial test (DESeq2, adjusted *P* < 0.05) and LDA effect size (LEfSe) analysis (67). Graphics were generated using R tools and GraphPad Prism software (version 9.0). Statistical significance was determined by analysis of variance (ANOVA) (details are provided in corresponding figure legends), *P* values <0.05 were deemed significant.

## Supplementary Material

Reviewer comments

## Data Availability

The 16S rRNA gene sequencing data generated in this study have been deposited in the National Center for Biotechnology Information (NCBI) Sequence Read Archive under the accession numbers PRJNA996099, PRJNA996786, and PRJNA996903.

## SUPPLEMENTAL MATERIAL

Supplemental figures msystems.00788-24-s0001.pdf Figures S1 to S6.

Supplemental tables msystems.00788-24-s0002.docx Tables S1 to S5.

OPEN PEER REVIEW reviewer-comments.pdf An accounting of the reviewer comments and feedback.

ASM does not own the copyrights to Supplemental Material that may be linked to, or accessed through, an article. The authors have granted ASM a non-exclusive, world-wide license to publish the Supplemental Material files. Please contact the corresponding author directly for reuse.
