# Supplementary material for: Targeted discovery of gut microbiome-remodeling compounds for the treatment of systemic inflammatory response syndrome
Source: mSystems. 2024 Sep 5;9(10):e00788-24. doi: 10.1128/msystems.00788-24 (PMC11494991; doi:10.1128/msystems.00788-24)
Supplement: Supplemental figures — Figures S1 to S6. [file msystems.00788-24-s0001.pdf]

Figure S1

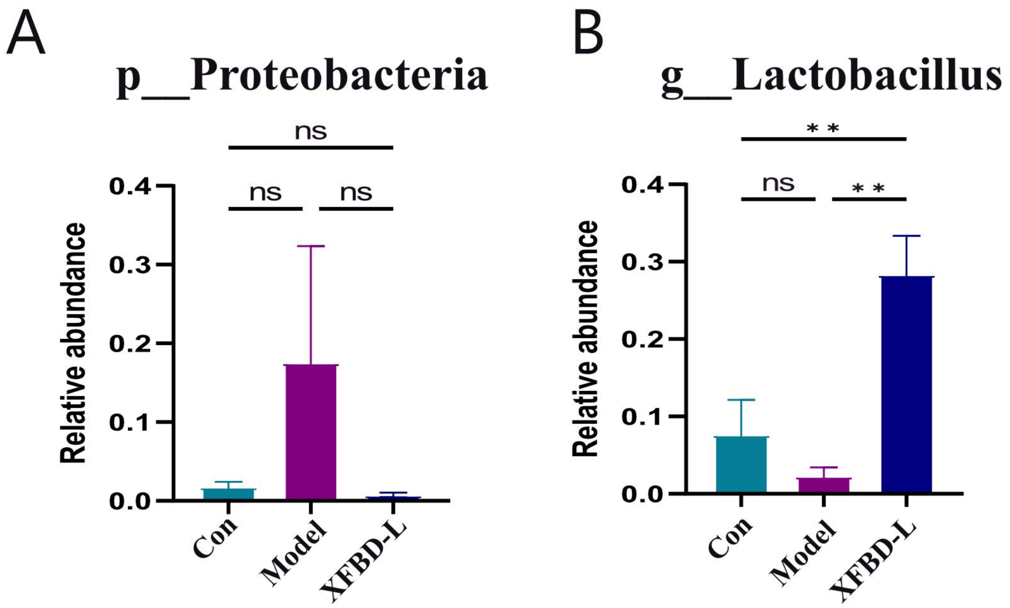

Fig. S1. Specific phylum and genus variations used for the XFBD modulates gut microbiome of LPS-induced SIRS mice in vivo. (A) The relative abundance of Proteobacteria in phylum level. (B) The relative abundance of Lactobacillus in genus level. Data are presented as mean ± SEM (n=5 per group). Statistical signifcance was determined using one-way ANOVA, followed by Tukey test. n.s. not significant,*P < 0.05, **P < 0.01, ***P < 0.001.

Figure S2

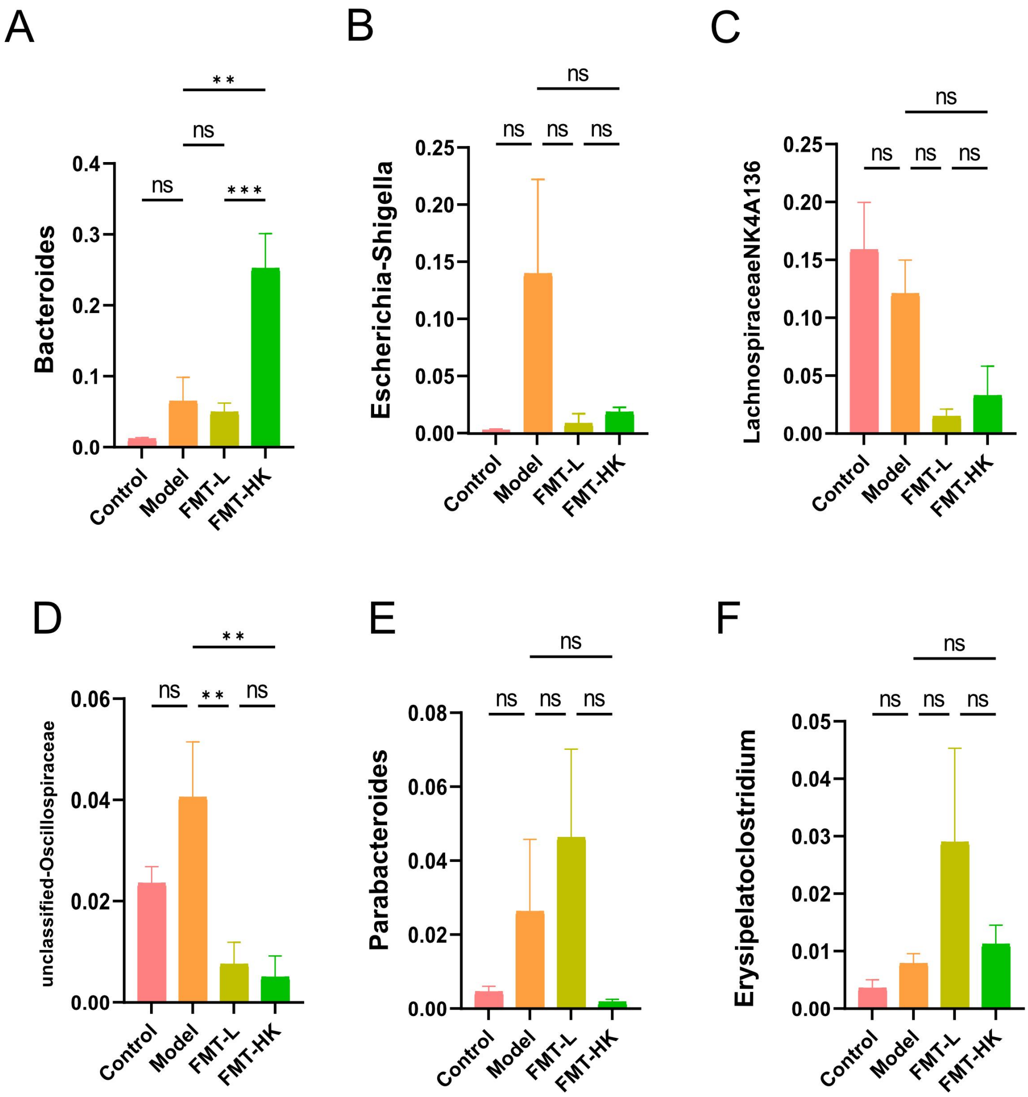

Fig. S2. Specific genus variations used for the FMT effects on microbiota in SIRS mice. The relative abundance of (A) Bacterodies, (B) Escherichia-Shigella, (C) LachnospiraceaeNK4A136, (D) unclassified-Oscillospiraceae, (E)Parabacteroides and (F)Erysipelatoclostridium in genus level. Data are presented as mean ± SEM (n=6 per group). Statistical signifcance was determined using one-way ANOVA, followed by Tukey test. n.s. not significant,*P < 0.05, **P < 0.01, ***P < 0.001，ns. not significant.

Figure S3

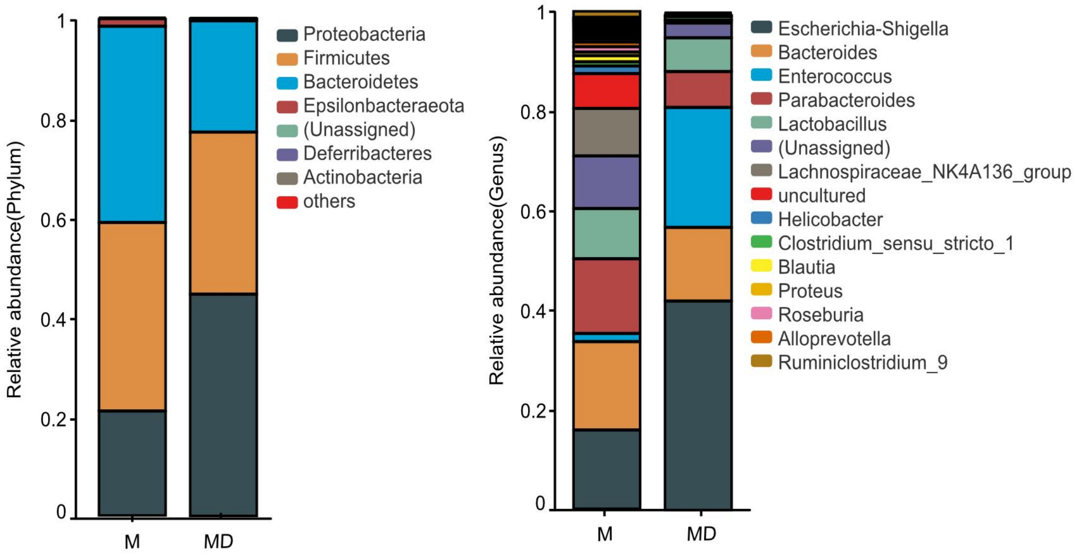

Fig. S3. Composition of the cultured gut bacteria community used for the in vitro screening of mono-compounds compared to the uncultured community. The major observed taxa at the phylum and genus levels are labeled on the right side of the figure.

Figure S4

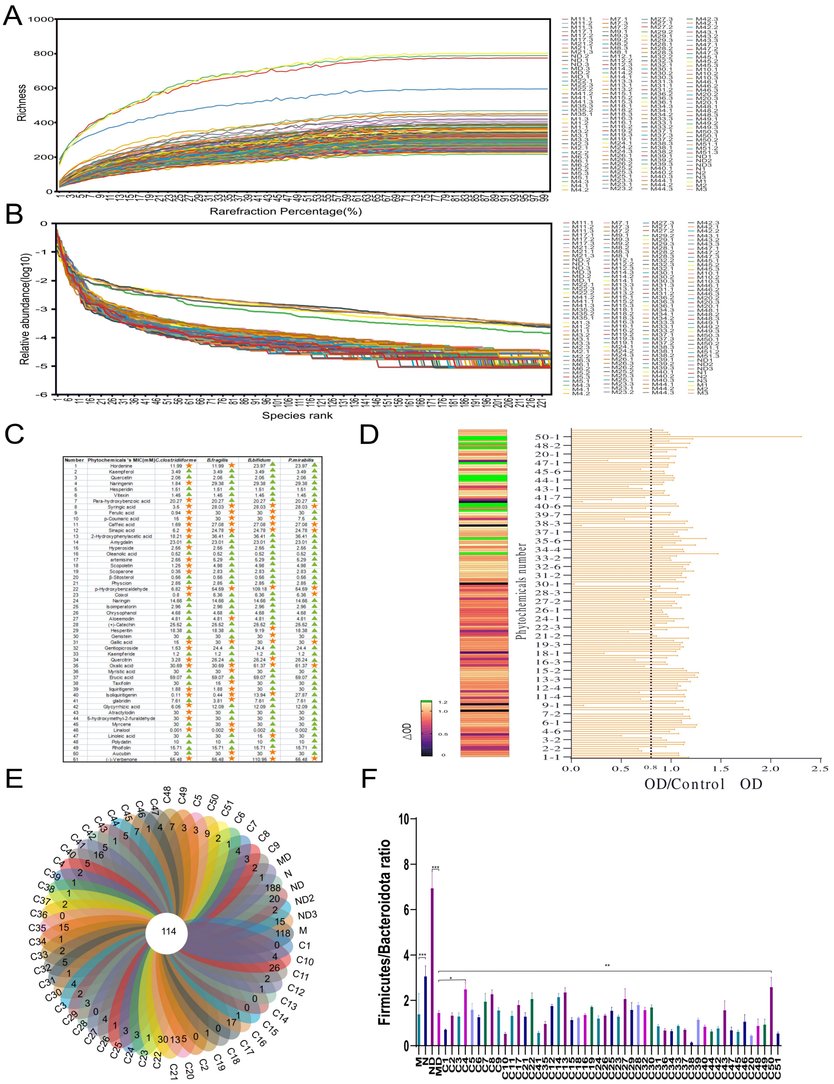

Number	Phytochemicals 's MIC(mM)	*C.clostridiiforme*	*B.fragilis*	*B.bifidum*	*P.mirabilis*
1	Hordenine	11.99 ★	11.99 ★	23.97 ▲	23.97 ▲
2	Kaempferol	3.49 ▲	3.49 ▲	3.49 ▲	3.49 ▲
3	Quercetin	2.06 ▲	2.06 ▲	2.06 ▲	2.06 ▲
4	Naringenin	1.84 ★	29.38 ▲	29.38 ▲	29.38 ▲
5	Hesperidin	1.51 ▲	1.51 ▲	1.51 ▲	1.51 ▲
6	Vitexin	1.45 ▲	1.45 ▲	1.45 ▲	1.45 ▲
7	Para-hydroxybenzoic acid	20.27 ★	20.27 ▲	20.27 ▲	20.27 ▲
8	Syringic acid	3.5 ★	28.03 ★	28.03 ★	28.03 ★
9	Ferulic acid	0.94 ★	30 ★	30 ★	30 ▲
10	p-Coumaric acid	15 ★	30 ★	30 ★	7.5 ▲
11	Caffeic acid	1.69 ★	27.08 ★	27.08 ★	27.08 ★
12	Sinapic acid	6.2 ★	24.78 ★	24.78 ★	24.78 ★
13	2-Hydroxyphenylacetic acid	18.21 ★	36.41 ▲	36.41 ▲	36.41 ▲
14	Amygdalin	23.01 ▲	23.01 ▲	23.01 ▲	23.01 ▲
15	Hyperoside	2.55 ★	2.55 ▲	2.55 ▲	2.55 ▲
16	Oleanolic acid	0.52 ▲	0.52 ▲	0.52 ▲	0.52 ▲
17	artemisine	2.65 ★	5.29 ▲	5.29 ▲	5.29 ▲
18	Scopoletin	1.25 ★	4.98 ▲	4.98 ▲	4.98 ▲
19	Scoparone	0.35 ★	2.83 ▲	2.83 ▲	2.83 ▲
20	β-Sitosterol	0.56 ▲	0.56 ▲	0.56 ▲	0.56 ▲
21	Physcion	2.85 ▲	2.85 ▲	2.85 ▲	2.85 ▲
22	p-Hydroxybenzaldehyde	6.82 ★	54.59 ★	109.18 ★	54.59 ★
23	Coixol	0.8 ★	6.36 ▲	6.36 ▲	6.36 ★
24	Naringin	14.66 ▲	14.66 ▲	14.66 ▲	14.66 ▲
25	Isoimperatorin	2.96 ▲	2.96 ▲	2.96 ▲	2.96 ▲
26	Chrysophanol	4.68 ▲	4.68 ▲	4.68 ▲	4.68 ▲
27	Aloeemodin	4.81 ▲	4.81 ★	4.81 ▲	4.81 ▲
28	(+)-Catechin	25.52 ▲	25.52 ▲	25.52 ▲	25.52 ▲
29	Hesperitin	18.38 ▲	18.38 ▲	9.19 ★	18.38 ▲
30	Genistein	30 ▲	30 ▲	30 ★	30 ▲
31	Gallic acid	15 ★	30 ★	30 ★	30 ★
32	Gentiopicroside	1.53 ★	24.4 ▲	24.4 ▲	24.4 ▲
33	Kaempferide	1.2 ▲	1.2 ▲	1.2 ▲	1.2 ▲
34	Quercitrin	3.28 ★	26.24 ▲	26.24 ★	26.24 ▲
35	Oxalic acid	30.69 ★	30.69 ★	61.37 ★	61.37 ★
36	Myristic acid	30 ▲	30 ★	30 ▲	30 ▲
37	Erucic acid	59.07 ▲	59.07 ▲	59.07 ▲	59.07 ▲
38	Taxifolin	30 ▲	15 ★	30 ▲	30 ▲
39	liquiritigenin	1.88 ★	1.88 ★	30 ▲	30 ▲
40	Isoliquiritigenin	0.11 ★	0.44 ★	13.94 ★	27.87 ▲
41	glabridin	7.61 ▲	3.81 ★	7.61 ▲	7.61 ▲
42	Glycyrrhizic acid	6.05 ★	12.09 ▲	12.09 ▲	12.09 ▲
43	Atractylodin	30 ★	30 ▲	30 ▲	30 ▲
44	5-hydroxymethyl-2-furaldehyde	30 ★	30 ▲	30 ▲	30 ▲
45	Myrcene	30 ▲	30 ★	30 ▲	30 ▲
46	Linalool	0.001 ★	0.002 ★	0.002 ▲	0.002 ▲
47	Linoleic acid	30 ▲	30 ▲	15 ★	30 ▲
48	Polydatin	10 ▲	10 ▲	10 ▲	10 ▲
49	Rhoifolin	15.71 ▲	15.71 ▲	15.71 ▲	15.71 ▲
50	Aucubin	30 ▲	30 ★	30 ★	30 ▲
51	(-)-Verbenone	55.48 ★	55.48 ★	110.95 ★	55.48 ★

Fig. S4. *In vitro* evaluation of mono-compounds on SIRS mice gut microbiota. The rarefaction curve (**A**)and rank abundance plot (**B**) of mice gut microbiota treated by compounds during in vitro screening. (**C**) Minimum inhibitory concentration (MIC) values for the screened mono-compounds against selected bacteria. The MIC was defined as the lowest concentration of monomer compounds that inhibited >90% of growth; lower MIC values correspond to greater antibacterial activity. The orange pentagram indicates the true inhibitory concentration. The green triangle indicates the concentration at which the mono-compound does not inhibit bacteria even at its maximum solubility in DMSO. (**D**)The change in OD of the whole gut microbiome before and after anaerobic culture at 37°C for 24 h. The heat map on the left shows the specific value of the change in OD, and the ratio of the treatment with different concentrations of mono-compounds to the control group is shown on the right. (**E**) Venn diagram of the composition of OTUs in cecal microbiota. (**F**) The ratio of Firmicutes/Bacteroidota in phylum levels.Data are presented as mean± SEM(n= 3 per group). Statistical signifcance was determined using one-way ANOVA, followed by Tukey test. n.s. not significant,*P < 0.05, **P < 0.01, ***P < 0.001, ns. not significant.

Figure S5

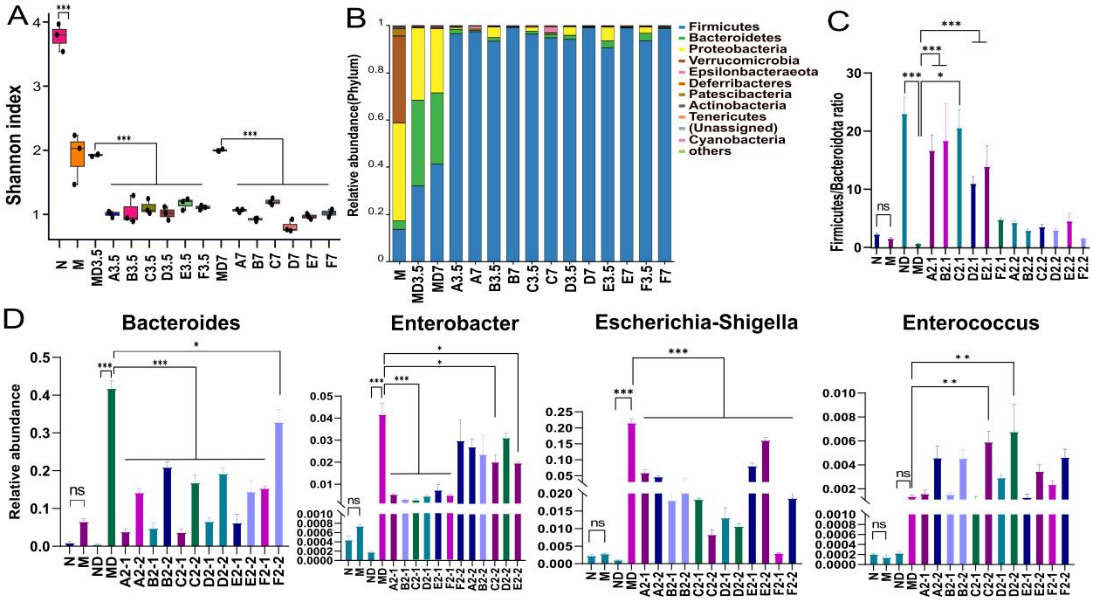

Fig. S5. (**A**) Shannon index of mice gut microbiota after treatment with high concentrations of GMRC cocktails. (**B**) Microbial composition at the phylum level after treatment with high concentrations of GMRC cocktails. (**C**) The ratio of Firmicutes/ Bacteroidota in phylum levels of GMRC cocktails. (**D**) The relative abundance of Bacteroides, Enterobacter, Escherichia-Shigella and Enterococcus in genus levels after cocktail treatment.Data are presented as mean ± SEM(n= 3 per group). Statistical signifcance was determined using one-way ANOVA, followed by Tukey test. n.s. not significant,*P < 0.05, **P < 0.01, ***P < 0.001, ns. not significant.

Figure S6

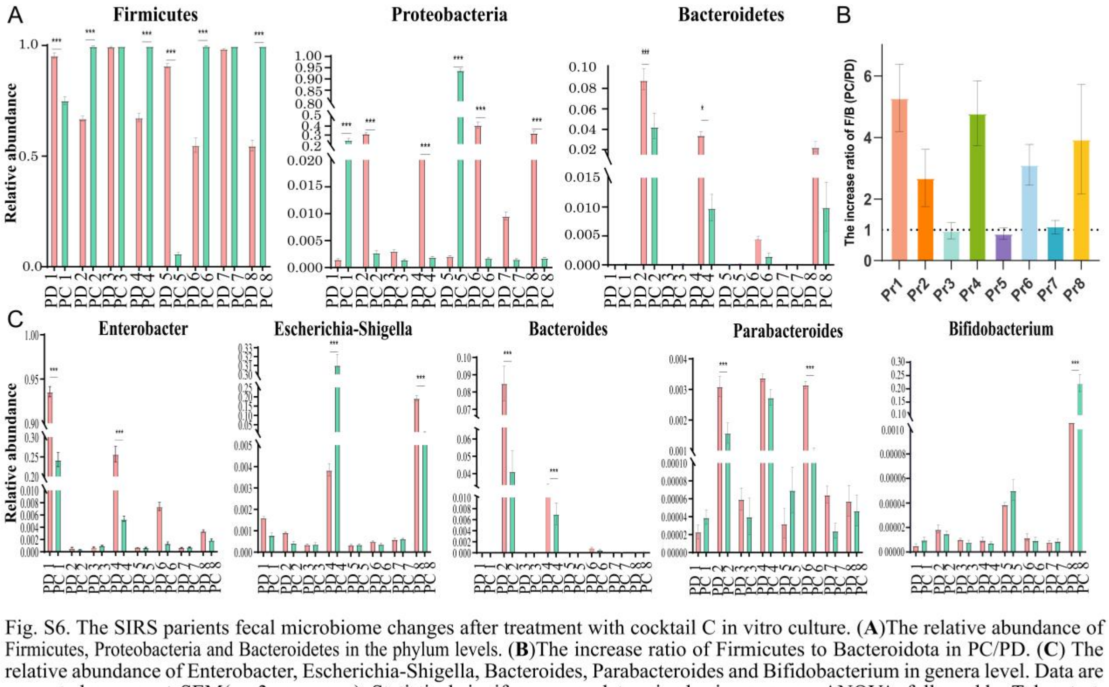

Fig. S6. The SIRS parients fecal microbiome changes after treatment with cocktail C in vitro culture. (**A**)The relative abundance of Firmicutes, Proteobacteria and Bacteroidetes in the phylum levels. (**B**)The increase ratio of Firmicutes to Bacteroidota in PC/PD. (**C**) The relative abundance of Enterobacter, Escherichia-Shigella, Bacteroides, Parabacteroides and Bifidobacterium in genera level. Data are presented as mean ± SEM(n= 3 per group). Statistical signifcance was determined using one-way ANOVA, followed by Tukey test. n.s. not significant,*P < 0.05, **P < 0.01, ***P < 0.001, ns. not significant.
