# Supplementary material for: Targeted discovery of gut microbiome-remodeling compounds for the treatment of systemic inflammatory response syndrome
Source: mSystems. 2024 Sep 5;9(10):e00788-24. doi: 10.1128/msystems.00788-24 (PMC11494991; doi:10.1128/msystems.00788-24)
Supplement: Supplemental tables — Tables S1 to S5. [file msystems.00788-24-s0002.docx]

Table S1. Detailed information of the compounds used in the study

| **Compounds** | **number** | **formula (M+H)** | **mass** | **purity** | **CAS** |
| --- | --- | --- | --- | --- | --- |
| Hordenine | 1 | C10H15NO | 165.23 | >98 | 539-15-1 |
| Kaempferol | 2 | C15H10O6 | 286.24 | >98 | 520-18-3 |
| Quercetin | 3 | C15H10O7 | 302.24 | >98 | 117-39-5 |
| Naringenin | 4 | C15H12O5 | 272.257 | >98 | 480-41-1 |
| Hesperidin | 5 | C28H34O15 | 610.56 | >98 | 520-26-3 |
| Vitexin | 6 | C21H20O10 | 432.38 | >98 | 3681-93-4 |
| Para-hydroxybenzoic acid | 7 | C7H6O3 | 138.12 | >99 | 99-96-7 |
| Syringic acid | 8 | C9H10O5 | 198.17 | >99 | 530-57-4 |
| Ferulic acid | 9 | C10H10O4 | 194.18 | >98 | 1135-24-6 |
| p-Coumaric acid | 10 | C9H8O3 | 164.16 | >98 | 501-98-4 |
| Caffeic acid | 11 | C9H8O4 | 180.16 | >98 | 331-39-5 |
| Sinapic acid | 12 | C11H12O5 | 224.21 | >98 | 530-59-6 |
| 2-Hydroxyphenylacetic acid | 13 | C8H8O3 | 152.15 | >98 | 614-75-5 |
| Amygdalin | 14 | C20H27NO11 | 457.43 | >98 | 29883-15-6 |
| Hyperoside | 15 | C21H20O12 | 464.38 | >98 | 482-36-0 |
| Oleanolic acid | 16 | C30H48O3 | 456.7 | >98 | 508-02-1 |
| artemisine | 17 | C15H22O5 | 282.33 | >99 | 63968-64-9 |
| Scopoletin | 18 | C10H8O4 | 192.17 | >99 | 92-61-5 |
| Scoparone | 19 | C11H10O4 | 206.19 | >98 | 120-08-1 |
| β-Sitosterol | 20 | C29H50O | 414.713 | >98 | 83-46-5 |
| Physcion | 21 | C16H12O5 | 284.26 | >98 | 521-61-9 |
| p-Hydroxybenzaldehyde | 22 | C7H6O2 | 122.12 | >99 | 123-08-0 |
| Coixol | 23 | C8H7NO3 | 165.148 | >98 | 532-91-2 |
| Naringin | 24 | C27H32O14 | 580.54 | >98 | 10236-47-2 |
| Isoimperatorin | 25 | C16H14O4 | 270.284 | >98 | 482-45-1 |
| Chrysophanol | 26 | C15H10O4 | 254.24 | >98 | 481-74-3 |
| Aloeemodin | 27 | C15H10O5 | 270.24 | >97 | 481-72-1 |
| (+)-Catechin | 28 | C15H14O6 | 290.27 | >98 | 154-23-4 |
| Hesperitin | 29 | C16H14O6 | 302.29 | >98 | 520-33-2 |
| Genistein | 30 | C15H10O5 | 270.24 | >98 | 446-72-0 |
| Gallic acid | 31 | C7H6O5 | 170.12 | >98 | 149-91-7 |
| Gentiopicroside | 32 | C16H20O9 | 356.32 | >98 | 20831-76-9 |
| Kaempferide | 33 | C16H12O6 | 300.267 | >98 | 491-54-3 |
| Quercitrin | 34 | C21H20O11 | 448.38 | >98 | 522-12-3 |
| Oxalic acid | 35 | C2H2O4 | 90.03 | >98 | 144-62-7 |
| Myristic acid | 36 | C14H28O2 | 228.37 | >98 | 544-63-8 |
| Erucic acid | 37 | C22H42O2 | 338.57 | >98 | 112-86-7 |
| Taxifolin | 38 | C15H12O7 | 304.25 | >98 | 480-18-2 |
| liquiritigenin | 39 | C15H12O4 | 256.26 | >98 | 41680-09-5 |
| Isoliquiritigenin | 40 | C15H12O4 | 256.257 | >98 | 961-29-5 |
| glabridin | 41 | C20H20O4 | 324.37 | >98 | 59870-68-7 |
| Glycyrrhizic acid | 42 | C42H62O16 | 822.93 | >98 | 1405-86-3 |
| Atractylodin | 43 | C13H10O | 182.22 | >98 | 55290-63-6 |
| 5-hydroxymethyl-2-furaldehyde | 44 | C6H6O3 | 126.112 | >98 | 67-47-0 |
| Myrcene | 45 | C10H16 | 136.236 | >95 | 123-35-3 |
| Linalool | 46 | C10H18O | 154.25 | >98 | 78-70-6 |
| Linoleic acid | 47 | C18H32O2 | 280.45 | >98 | 60-33-3 |
| Polydatin | 48 | C20H22O8 | 390.39 | >98 | 27208-80-6 |
| Rhoifolin | 49 | C27H30O14 | 578.526 | >98 | 17306-46-6 |
| Aucubin | 50 | C15H22O9 | 346.333 | >98 | 479-98-1 |
| (-)-Verbenone | 51 | C10H14O | 150.22 | >99 | 1196-01-6 |

**Table S2** The constituent and dose of GMRC-cocktails used to treat SIRS mice

| **Compound number** | **Cocktails** | | | **Dose(mg/kg/d)** |
| --- | --- | --- | --- | --- |
|  | **C.v1** | **C.v2** | **C.v3** |  |
| 50 | 50 | 50 | - | 50 |
| 32 | 32 | - | - | 40.5 |
| 8 | 8 | 8 | 8 | 11.5 |
| 31 | 31 | 31 | 31 | 50 |
| 22 | 22 | 22 | 22 | 91 |
| 7 | 7 | 7 | 7 | 34 |
| 25 | 25 | 25 | 25 | 5 |

**Table S3** Patients’ characteristics

| **Patients’ characteristics** | **Patients enrolled** | **Controls** |
| --- | --- | --- |
| Number | 8 | 10 |
| Age [years] | 55.2 (42-68) | 53 (50-65) |
| Gender [male/female] | 4/4 | 5/5 |
| BMI [kg/m2] | 23.78 (19.6-25.4) | ND |
| Diagnosis [n (%)] | Sepsis: n = 8 (100) | NA |
| Severity of illness | ICU scores: | NA |
| (at ICU admission) | SOFA: 6.75 (6-8) |  |
|  | APACHE-II: 22.25 (21-25) |  |

BMl, body mass index;

ICU, intensive care unit;

NA, not applicable;

SOFA, Sequential Organ Failure Assessment score;

APACHE-II, Acute Physiology and Chronic Health Evaluation II.

**Table S4** List of bacterial taxa observed in uncultured cecum samples of C57BL/6 mice and in the in vitro GMRC screening assay

| **Taxon** | **Present within *in vitro* culture?** | **Taxon** | **Present within *in vitro* culture?** |
| --- | --- | --- | --- |
|  |  |  |  |
|  |  |  |  |
|  |  |  |  |
|  |  |  |  |
| g_ Anaerostipes | – | g_Rikenellaceae_RC9_gut_group | + |
| g_Ruminococcus_1 | – | g_Parasutterella | + |
| g_Ralstonia | – | g_Erysipelatoclostridium | + |
| g_Cupriavidus | – | g_Lachnospiraceae_UCG-006 | + |
| g_Turicibacter | – | g_Ruminiclostridium | + |
| g_Ruminiclostridium_6 | – | g_Lachnospiraceae_UCG-001 | + |
| g_Bacillus | – | g_Ruminococcaceae_UCG-014 | + |
| g_Ruminococcaceae_UCG-005 | – | g_Anaerotruncus | + |
| g_Sphingomonas | – | g_GCA-900066575 | + |
| g_Delftia | – | g_Sutterella | + |
| g_Acetitomaculum | – | g_Marvinbryantia | + |
| g_Candidatus_Soleaferrea | – | g_Muribaculum | + |
| g_Streptococcus | – | g_Desulfovibrio | + |
| g_Acinetobacter | – | g_UBA1819 | + |
| g_Family_XIII_AD3011_group | – | g_Bifidobacterium | + |
| g_Dubosiella | – | g_Ruminiclostridium_5 | + |
| g_Coriobacteriaceae_UCG-002 | – | g_Faecalibacterium | + |
| g_Anaeroplasma | – | g_Tyzzerella | + |
| g_Escherichia-Shigella | + | g_Butyricicoccus | + |
| g_Bacteroides | + | g_Enterorhabdus | + |
| g_Lactobacillus | + | g_Lachnospiraceae_FCS020_group | + |
| g_Enterococcus | + | g_Ruminococcaceae_UCG-009 | + |
| g_Parabacteroides | + | g_Allobaculum | + |
| g_Proteus | + | g_Stenotrophomonas | + |
| g_Clostridium_sensu_stricto_1 | + | g_Achromobacter | + |
| g_Lachnospiraceae_NK4A136_group | + | g_Negativibacillus | + |
| g_Helicobacter | + | g_Prevotellaceae_UCG-001 | + |
| g_Blautia | + | g_Candidatus_Saccharimonas | + |
| g_Alloprevotella | + | g_A2 | + |
| g_Pseudomonas | + | g_Ruminococcaceae_NK4A214_group | + |
| g_Roseburia | + | g_Harryflintia | + |
| g_Ruminiclostridium_9 | + | g_Family_XIII_UCG-001 | + |
| g_Lachnoclostridium | + | g_Peptococcus | + |
| g_ASF356 | + | g_Akkermansia | + |
| g_Asaccharospora | + | g_Ruminococcaceae_UCG-010 | + |
| g_Butyricimonas | + | g_Staphylococcus | + |
| g_Bilophila | + | g_Eisenbergiella | + |
| g_Alistipes | + | g_Terrisporobacter | + |
| g_Oscillibacter | + | g_Christensenellaceae_R-7_group | + |
| g_Mucispirillum | + | g_Lachnospiraceae_UCG-004 | + |

**Table S5** The number OTUs of different treatment groups

| **SampleID** | **No. of OTUs** | **No. of seqs** |
| --- | --- | --- |
| N | 1040 | 292225 |
| M | 965 | 282119 |
| ND | 623 | 328685 |
| MD | 380 | 318052 |
| C1 | 362 | 330413 |
| C2 | 393 | 330803 |
| C3 | 381 | 333572 |
| C4 | 427 | 331877 |
| C5 | 415 | 310166 |
| C6 | 385 | 303334 |
| C7 | 411 | 307149 |
| C8 | 386 | 331909 |
| C9 | 416 | 324531 |
| C10 | 483 | 324831 |
| C11 | 549 | 685832 |
| C12 | 392 | 316543 |
| C13 | 384 | 314491 |
| C14 | 354 | 323774 |
| C15 | 399 | 320131 |
| C16 | 386 | 332243 |
| C17 | 461 | 677503 |
| C18 | 395 | 320047 |
| C19 | 437 | 327755 |
| C20 | 476 | 325152 |
| C21 | 463 | 648166 |
| C22 | 632 | 658648 |
| C23 | 425 | 318251 |
| C24 | 397 | 323990 |
| C25 | 408 | 335841 |
| C26 | 419 | 334296 |
| C27 | 421 | 330813 |
| C28 | 413 | 344872 |
| C29 | 430 | 325817 |
| C30 | 394 | 327613 |
| C31 | 370 | 329545 |
| C32 | 397 | 324971 |
| C33 | 371 | 330325 |
| C34 | 384 | 338237 |
| C35 | 533 | 688910 |
| C36 | 373 | 333838 |
| C37 | 386 | 333884 |
| C38 | 396 | 324257 |
| C39 | 350 | 326301 |
| C40 | 493 | 332144 |
| C41 | 505 | 673656 |
| C42 | 508 | 333375 |
| C43 | 457 | 337141 |
| C44 | 451 | 340165 |
| C45 | 452 | 307696 |
| C46 | 457 | 313135 |
| C47 | 434 | 304129 |
| C48 | 579 | 316293 |
| C49 | 493 | 317812 |
| C50 | 553 | 318173 |
| C51 | 459 | 319895 |
